# Release of Adenosine and ATP During Ischemia and Epilepsy

**DOI:** 10.2174/157015909789152146

**Published:** 2009-09

**Authors:** Nicholas Dale, Bruno G Frenguelli

**Affiliations:** Department of Biological Sciences, University of Warwick, Coventry, CV4 7AL, UK

## Abstract

Eighty years ago Drury & Szent-Györgyi described the actions of adenosine, AMP (adenylic acid) and ATP (pyrophosphoric or diphosphoric ester of adenylic acid) on the mammalian cardiovascular system, skeletal muscle, intestinal and urinary systems. Since then considerable insight has been gleaned on the means by which these compounds act, not least of which in the distinction between the two broad classes of their respective receptors, with their many subtypes, and the ensuing diversity in cellular consequences their activation invokes. These myriad actions are of course predicated on the release of the purines into the extracellular milieu, but, surprisingly, there is still considerable ambiguity as to how this occurs in various physiological and pathophysiological conditions. In this review we summarise the release of ATP and adenosine during seizures and cerebral ischemia and discuss mechanisms by which the purines adenosine and ATP may be released from cells in the CNS under these conditions.

## INTRODUCTION

It is now beyond dispute that purine compounds such as adenosine and ATP are released from cells of the mammalian central nervous system and exert powerful actions on neuronal function *via* a variety of cell surface receptors. To have arrived at this conclusion required work spanning eight decades. In this review we provide an account of the studies that demonstrated the release of purine compounds following physiological or pathological stimulation of brain tissue *in vitro* and *in vivo* and describe some of the possible conduits of purine release under these conditions. Although there is evidence for guanine nucleotide release in the central nervous system, most likely from glial cells, and extracellular conversion to guanosine [167], we shall restrict this review to adenosine and ATP. Molecular aspects of purinergic signalling are not covered in detail, but can be found in several excellent recent reviews [2,3,53,88,96,170,207,231], whilst a more systems-level understanding of the role of purines in the CNS can be found in other chapters of this Hot Topics issue of Current Neuropharmacology.

## ADENOSINE AND ATP AS NEUROMODULATORS

The possibility that the purines adenosine and ATP could influence neuronal function in the mammalian CNS was tantalizingly hinted at in Alan Drury & Albert Szent-Györgyi's seminal study [44] during which they reported that "The animal [guinea pig] immediately after the [sc] injection [of adenosine] appears normal, but within a minute or two develops a panting respiration, becomes listless, and tends to lie quite still and on occasions to sleep.".

However, these scientists, one of whom (Drury) went on to become elected Fellow of the Royal Society (1937) and receive a Knighthood (1950), and the other to receive a Nobel Prize (Szent-Györgyi; Physiology or Medicine, 1937), could not state "…whether an action on the nervous system must be considered…" as responsible for the adenosine-induced somnolence, given the profound effects on blood pressure and heart rate. Fast forward eighty years and the role of adenosine in sleep is firmly established [75,109] (see also Greene, this issue).

The realisation that purines were major players in the CNS was hard won in the ensuing decades and emerged slowly in the 1960 and 70s after a series of largely negative findings related to the actions of iontophoretically-applied ATP to neurones in cortex and spinal cord [30,104]. This was in contrast to work in the periphery, spearheaded by Geoff Burnstock, which was more supportive of a role for ATP as a potential neurotransmitter [20,79], although the concept that precious ATP, the cellular energy currency, might be released from cells under normal circumstances was heretical and fiercely resisted [21]. Subsequent work by John Phillis and colleagues indicated that ATP and adenine derivatives such as adenosine were powerful suppressors of neuronal firing in the brain [156,157], an action we now attribute to the dominant role of the adenosine A_1_ receptor.

These early studies paved the way for what we now know that adenosine and ATP exert their unequivocal neurotransmitter and neuromodulator actions, both excitatory and inhibitory, in the mammalian CNS *via* a large variety of purine receptors. These are divided into the P1 and P2 receptors. P1 receptors are activated primarily by adenosine, but some (eg A_3_R) may also be activated by the immediate metabolite of adenosine, inosine. Adenosine receptors, of which four are presently known - A_1_, A_2A_, A_2B_ and A_3_, are all 7-transmembrane spanning G-protein-coupled receptors with A_1_ and A_3_ primarily coupling to G_i/o_ and the A_2_ receptors coupling to G_s/olf_ [22,64,165]. It is through these receptors that adenosine modulates or "fine-tunes" synaptic transmission in the CNS (see Sebastião, this issue).

P2 receptors are sub-divided into P2X and P2Y groups [25]. P2X receptors are trimeric ligand-gated ion channels and presently comprise seven subtypes (P2X_1-7_), activated by ATP [88,96,170]. P2X receptors form functional receptors in various homo- and hetero-oligomeric assemblies of individual P2X subunits and all P2X channels are permeable to Na^+^, K^+^ and Ca^2+^. However some are permeable to Cl^-^, whilst under certain conditions some subtypes undergo pore dilation and become permeable to larger molecules such as NMDG^+^, YO-PRO, glutamate and even ATP [53].

P2Y receptors, like P1 receptors are 7-transmembrane spanning G-protein-coupled receptors of which there are eight subtypes (P2Y_1,2,4,6,11,12,13,14_). The P2Y nomenclature lacks several members, reflecting either the existence of nonmammalian orthologs or receptors having some P2Y receptor sequence similarity but no apparent response to nucleotides. These receptors are designated as p2y [2].

In the case of P2Y receptors the nature and order of potency for nucleotides differs and includes the purines ATP, ADP, and the pyrimidines, UTP, UDP and UDP glucose as agonists at human P2Y receptors. [2]. A subdivision of P2Y receptors has been proposed based on phylogenetic and structural similarities [1], one group comprising P2Y_1,2,4,6,11_ and another containing P2Y_12,13,14_. Interestingly heteromers exist between P2Y_1_ and A_1_ [225-227] and P2Y_2_ and A_1_ receptors [196], whilst functional interactions between P2Y_1_ and A_1_ are found in the brain [200,201]. These heteromers suggests a relationship between adenosine and ATP more intimate than that predicted from one simply being a metabolite of the other.

**Fig. (1):**
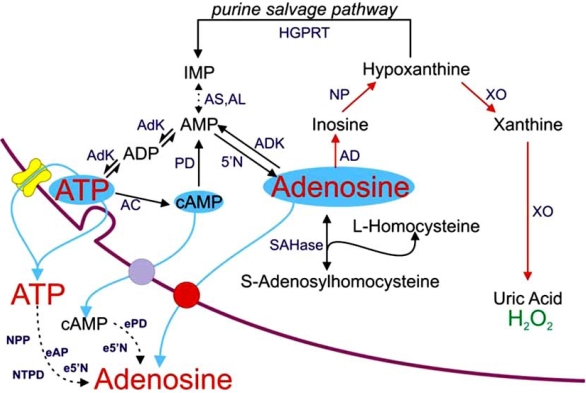
Sources of extracellular adenosine and ATP. 5'N, cytosolic 5'-nucleotidase; AC, adenylate cyclase; AD adenosine deaminase; AdK, adenylate kinase; ADK, adenosine kinase; AL, adenylosuccinate lyase; AS, adenylosuccinate synthase; e5'N, ecto 5'-nucleotidase; eAP, ecto-alkaline phosphatase; ePD, ecto-phosphodiesterase; HGPRT, hypoxanthine phospho-ribosyl-transferase; NP, nucleoside phosphorylase; NPP, nucleotide pyrophosphatase/phosphodiesterase; NTPD, nucleoside triphosphate diphosphohydrolase; SAHase, S-adenosyl-L-homocysteine hydrolase; XO, xanthine oxidase. In red is the primary route of adenosine metabolism and the enzymic cascade utilised by the adenosine biosensor. Modified from [148].

The actions of ATP and adenosine at these extracellular receptors are critically dependent on the release of these purines from cells. That this is self evident is obvious, but surprisingly, the release process remains mysterious in many instances and likely reflects the complexity of purine production, metabolism and release (Fig. **1**). In this review we first describe the release of adenosine during hypoxia/ischemia and seizure activity, and then discuss the more recently described ways in which these purines can be released.

## EARLY STUDIES OF PURINE RELEASE

The possibility that purines may be released from the CNS emerged in a flurry of activity in the late 1960s and early 1970s, primarily led by Henry McIlwain. These early studies involved the incorporation and release of ^14^C-labelled adenine-containing compounds, usually adenine and adenosine.

In a pivotal study of 1969, Kakiuchi, Rall and McIlwain [91] showed, for the first time, that electrical stimulation of guinea pig cerebral cortex resulted in a large (11 fold) increase in tissue cAMP, a compound, which due to the seminal and ultimately 1971 Nobel Prize winning work of Earl Sutherland, in collaboration with Ted Rall, in the late 50s and early 60s [166,195] had garnered a great deal of interest in the neuroscience community. The increase in cAMP was greatly attenuated by the adenosine receptor antagonist theophylline, in a manner related to the duration of electrical stimulation: theophylline had greater effect against shorter durations of stimulation, a finding we would now interpret as due to the competitive nature of theophylline against adenosine receptors. This work led the authors to propose a mechanism for the elevation of cAMP in which "….electrical pulses exerted their effects by causing the release of active endogenous organic substances." and that further studies would "…be conducted to test the possibility that the effects of electrical pulses involves the release and action of adenine-ribose compounds." In the classic Sattin and Rall study of 1970 they conclusively showed that exogenous adenosine was capable of stimulating the production of cAMP and that methylxanthines could antagonise this effect [180] strengthening the possibility that adenosine might be released in response to electrical stimulation.

An indication that these prescient statements regarding the release of purines were to come to fruition was obtained the following year in a paper from John Daly's lab [186] in which they observed release of adenine-^14^C derived radioactivity into the medium surrounding guinea pig cortical brain slices. Adenosine was found in greatest quantities (~50 % of the radioactivity) with contributions from hypoxanthine and inosine. Importantly, the release of adenosine was increased by elevated K^+^, veratridine and ouabain. Indeed, veratridine also permitted the detection of small amounts of adenine nucleotides in the medium. The authors, based on a proposal made a few years earlier by Sattin & Rall, suggested that depolarisation induced the release of adenosine and that this adenosine then stimulated the production of cAMP.

However, it was not until the following year that Henry McIlwain reported the release of adenosine, inosine and hypoxanthine by electrical stimulation of brain slices and led to the proposal of "Synaptic sites of adenosine action?" [130, 131]. These were followed by a series of full reports further characterising this observation. The first of these [162] showed that the ^14^C-labelled compounds released, in a stimulation-dependent manner, contained mostly adenosine, but some inosine, hypoxanthine and nucleotides. This paper also showed that oxygen or glucose deprivation also caused the efflux of ^14^C-labelled derivatives of adenine, which, by extension to their work with derivatives released by electrical stimulation, would likely have included adenosine. The observations in this paper on adenosine release, and the appreciation of the wider context and implications of these findings in "coma accompanying hypoglycaemia", "convulsive episodes" and "the neural threshold for excitation" prompted the authors to conclude that "Intermediation by adenosine in these various processes merits investigation." Some 37 years later, this volume testifies that this prediction has certainly been fulfilled.

Subsequent studies from the McIlwain lab established many of the key features of adenosine release that still hold sway. For example, his lab noted [163] that basal adenosine release was augmented in Ca^2+^-free medium, but that evoked by electrical stimulation was almost abolished by Ca^2+^-free medium and substantially inhibited by TTX; that release could be increased by preventing reuptake (with 2'-deoxyadenosine and papaverine); inferred that adenosine kinase was a major regulator of adenosine release and, on the basis of release of adenine derivatives per stimulus being similar to that described for ATP by Geoff Burnstock at peripheral synapses [24], predicted that cerebral adenine nucleotides could be released in response to electrical stimulation [130,161,163]. That adenosine and ATP release could be evoked from the intact cerebral cortex by electrical stimulation, as demonstrated by John Phillis, indicated that such release was not a phenomenon restricted to isolated tissue preparations [194,223].

The slew of ensuing studies by many other groups on the release of brain adenosine and ATP evoked by additional stimuli such as excitatory amino acids and other transmitters, including the methods used to collect and analyse samples, have been reviewed by others and provide valuable historical and more contemporary accounts [22,112,154,158,215].

## RELEASE OF PURINES DURING METABOLIC STRESS

Evidence obtained by Bob Berne in the early 70s indicated that adenosine accumulated in cardiac and skeletal muscle in response to ischemia. The first description of release of adenosine from the *in vivo* brain during ischemia also came from his lab [8]. In this study he and his colleagues showed that global cerebral ischemia caused an increase in tissue adenosine (as shown earlier by Deuticke and Gerlach in the rat [37]) in both dog and rat brain. Importantly, they also observed release of adenosine, and the metabolites inosine and hypoxanthine, into the cerebrospinal fluid after 6 mins of cerebral ischemia. In attempting to ascribe a role to this adenosine release they showed that injection of adenosine into the carotid arteries increased cerebral blood flow in the dog to a small extent whilst topical exogenous adenosine caused marked vasodilatation of pial arterioles in dog and cat (where it was easier to quantify), but injection did not, most likely reflecting breakdown or uptake of exogenous adenosine. It is interesting to note that the vexed question of whether adenosine arises from "….neural cells, glial cells, or from both…" was asked even then.

## PURINE SENSING - THE NEED FOR SPEED

An appreciation that changes in the extracellular concentration of purines might influence physiological activity on the order of seconds prompted the Berne lab to adopt the innovative "freeze-blowing" technique devised by Veech *et al.,* [205] in which high pressure air is blown into one side of the cranial cavity *via* a sharp hollow steel probe and the supratentorial brain expelled *via* another tube on the contralateral side onto a liquid N_2_-cooled aluminium plate, thus immediately halting metabolic reactions at defined time points on the order of seconds after an intervention.

With this technique Dick Winn *et al.,* [219] were able to discern changes in tissue adenosine with 5 s of global cerebral ischemia following aortic transaction. This allowed the authors to conclude that changes in tissue adenosine may be sufficiently rapid to regulate cerebral blood flow, a conclusion that has now been widely accepted [70,106,154].

Of course, cerebral vasodilatation is not the only rapid consequence of brain hypoxia/ischemia. It has been know for many years that cortical electrical activity is rapidly suppressed by such insults (reviewed by [103]). Early indications that the suppression of glutamatergic excitation during hypoxia/ischemia might occur through the release of adenosine emerged following the seminal studies of John Phillis, who showed that exogenous adenosine inhibited cortical and cerebellar activity *in vivo* [102,157] and, with the limitations of the drugs and techniques available at the time, observed some evidence *in vivo* of excitation by adenosine antagonists caffeine and theophylline [155].

Accordingly, Tom Dunwiddie [48] showed conclusively that *endogenous* adenosine could exert effects on synaptic transmission in the *in vitro* hippocampus by demonstrating that application of both adenosine deaminase, to metabolise extracellular adenosine to inosine, and the adenosine receptor antagonists theophylline and IBMX increased field excitatory postsynaptic (fEPSP) and population spikes, whilst the adenosine uptake inhibitor hexobendine inhibited transmission. Hexobendine had been previously shown by the Daly lab, to elevate tissue cAMP levels, indicative of both an extracellular adenosine tone and adenosine receptors [80].

We now know that these depressant actions of adenosine are due to the activation of presynaptic adenosine A_1_ receptors [16,49,89]. Subsequent work from Brian MacVicar's lab suggests that this inhibition of glutamatergic transmission involves activation of p38 MAPK cascade [19] and C-Jun N-terminal kinase [18]. The extent to which this pathway impacts on the known adenosine-mediated inhibition of Ca^2+^ spikes [160] and N- and P/Q-type Ca^2+^ channels [14,73, 134,222,224], which is at least in part membrane-delimited and requires βγ subunits, is not yet clear.

Putting the two together - release of adenosine during hypoxia and the inhibition of excitatory synaptic transmission by hypoxia - did not come about until the early 1980s when a Society for Neuroscience abstract appeared from Lipton and Robacker [115,117] indicating that the hypoxic depression of hippocampal dentate gyrus population spikes was slowed by both IBMX and adenosine deaminase (both of which increased the population spike under control conditions). This was not due to better maintenance of tissue ATP levels, which had been shown to delay the hypoxic depression of transmission [116]. In contrast IBMX caused a greater decline in tissue ATP, most likely *via* greater continued glutamatergic excitation during the hypoxic episode.

A delay in the hypoxic depression of hippocampal fEPSPs by 8-PT was later observed by Dunwiddie and Bertil Fredholm, who also observed a late (14 min) rise in adenosine release during hypoxia as measured by HPLC analysis of perfusate [63]. This delay in efflux *vs* effects on synaptic transmission has been observed by others using HPLC techniques [59,60,153], but this is likely due to low temporal resolution of earlier HPLC studies. Better (2 min) resolution showed a clearer correlation between the two [110].

However, by this time, several groups had convincingly shown pharmacologically that the depression of synaptic transmission caused by hypoxia and combined oxygen/glucose deprivation (OGD, "*in vitro* ischemia"), in spinal cord [122] and in hippocampus [57,58,71], as well as the depression caused by hypoglycaemia [228] and cyanide [229], were dependent on adenosine release and activation of A_1_Rs. This has been subsequently confirmed *in vivo* for hypoxia/ ischemia [69].

Adenosine thus inhibits glutamate release during metabolic stress and does not need glutamate receptor activation for its release [65,66,149]. It would seem that adenosine may be released prior to the release of glutamate and could thus delay potentially excitotoxic glutamate release. Indeed, *in vivo* it is clear that this occurs: adenosine is released from the cat brain at blood flow levels (~25 ml/100 g/min) higher than flow levels required for glutamate release (~20 ml/100 g/min) [128]. In between this range the electrocorticogram (ECoG) was reduced by 80 %, suggestive of a major role for adenosine in ECoG suppression. This has recently been confirmed *via* the use of A_1_R antagonists which delayed the ischemic suppression of the ECoG [83].

These observations satisfy Andrew Newby's concept of adenosine as a retaliatory metabolite, which he described in the following terms: "Physiologically, adenosine's function appears to be to allow a target cell such as the cardiac myocyte to adjust its energy supply and to retaliate against an external stimulus which would tend to cause excessive ATP breakdown." [138]. It is now clear that not only does adenosine retaliate, it gets its retaliation in first!

## ENZYME-BASED ADENOSINE SENSORS - SENSING IN THE FAST LANE

**Fig. (2):**
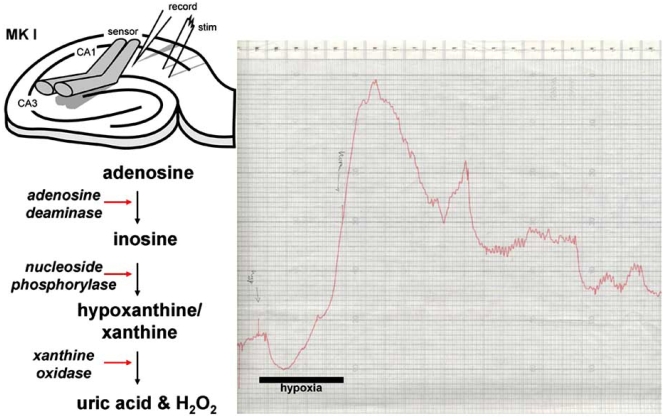
Principle behind the MK I adenosine sensor. One barrel of the sensor contains the full complement of enzymes, whilst a second lacks adenosine deaminase. The right panel shows the original chart recording of the second ever sensor recording of adenosine release in response to a 5 min period of hypoxia. The first recording went off scale!

Improved correlations between adenosine release during hypoxia/ischemia and the inhibition of excitatory synaptic transmission have been made possible through the use of adenosine biosensors, which allow direct, real-time correlations between the two. The first generation biosensors [31] relied upon a solution of adenosine metabolising enzymes (adenosine deaminase, purine nucleoside phosphorylase and xanthine oxidase) housed within semipermeable microdialysis tubing into which adenosine could diffuse following release from tissue (Fig. **2**). The metabolism of adenosine gave rise to hydrogen peroxide which is oxidised on polarised Pt or Pt/Ir wire to produce a current proportional to the initial concentration of adenosine. Two such sensors, one with the full complement of enzymes and one lacking adenosine deaminase, to provide an "inosine" signal, are used in parallel and a differential recording between the two yields a specific adenosine signal, which can both be quantified (*via* calibration with exogenous adenosine) and confirmed through the use of an adenosine deaminase inhibitor such as EHNA or coformycin.

The first application of these sensors to mammalian tissue involved them being placed on the surface of hippocampal slices [34] (Fig. **2**). During the onset of hypoxia, after a brief transient negative deflection, a steadily rising signal was observed on the sensors, which correlated well with the depression of the simultaneously-recorded fEPSP, and scaled with the duration of hypoxia. Reintroduction of oxygenated aCSF caused a decline in the adenosine signal to baseline levels. Comparison of the hypoxic fEPSP depression and the levels of adenosine as measured at the surface of the slice yielded an IC_50_ of around 3 μM. In this study, the removal of Ca^2+^ from the aCSF resulted in an increase in hypoxic adenosine release, confirming McIlwain's earlier observation [163], and those of others in hippocampal slices [153] and cultured astrocytes [125]. In contrast, elevating extracellular Ca^2+^ reduced both adenosine release and delayed the hypoxic depression of the fEPSP.

The basis of this inverse relationship between extracellular Ca^2+^ and adenosine release is not clear, but may reflect some Ca^2+^-dependency of adenosine release or metabolic processes. Nonetheless, it implies that at times when extracellular Ca^2+^ is reduced through hypoxia/ischemia [127] or in an activity-dependent manner [189], adenosine release is facilitated. This has implications for neuroprotection, the observed increase in the tone and influence of adenosine following repetitive synaptic activity [210] and potentially in the adenosine-based suppression of seizure activity [46,54].

## ADENOSINE RELEASE - A LABILE PROCESS

Given the importance of adenosine as a neuroprotective agent [29,175] (see contributions by Williams, Lusardi & Masino, this issue), although this may be developmentally-regulated [97,202,213], it is somewhat surprising to note that the availability of adenosine is not fixed, but labile and dependent on prior adenosine release [148]. This was first described in Fredholm's lab [51] where carotid artery occlusion in the gerbil resulted in large elevations in response to the first 5 min occlusion, but smaller rises in response to the second. Similar observations were made in the rat in response to two 10 mins periods of increased intracranial pressure separated by two hours: the release of cortical adenosine, inosine, hypoxanthine and xanthine were reduced during the second [204].

One functional implication of this adenosine depletion emerged from a study relating adenosine release to cerebral blood flow: in piglets subjected to bicuculline-induced seizures, hypoxia was less effective at inducing cerebral vasodilatation, which could be restored by topical application of exogenous adenosine, at non-dilating concentrations, suggesting the loss of adenosine during the seizure [38]. That adenosine is released during seizure activity is discussed later, but the important point here is that the "pool" of adenosine recruited (and depleted) by seizure activity and hypoxia/ischemia are similar. Furthermore, adenosine release in the nucleus tractus solitarius induced by sequential stimulation (100 Hz for 5s) of the hypothalamic defence area resulted in both reduced adenosine release (as measured with biosensors) and faster recovery of phrenic nerve activity, indicative of reduced apnoea [33]. This observation may underlie the plasticity in behavioural, respiratory and cardiovascular components of the hypothalamic defence response [126].

**Fig. (3):**
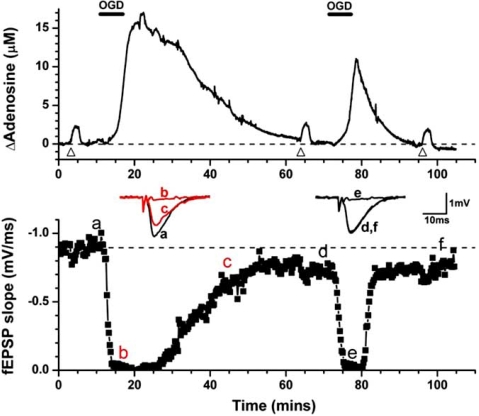
Repeated *in vitro* ischemia results in reduced adenosine release in hippocampal slices. Upper trace - adenosine release as measured by a MK I sensor in response to two sequential periods of oxygen/glucose deprivation (OGD; black bars). Bottom trace - depression and recovery of synaptic transmission in response to the OGD episodes. Note the reduced release of adenosine and reduced effects on the fEPSP during the second period. Inset are individual fEPSPs taken at the times indicated. Triangles refer to applications of exogenous adenosine to test that the sensor has not run down over this period.

Subsequent studies in *in vitro* hippocampal slices observed similar reductions in extracellular adenosine release during repeated hypoxia [65,151,152] and OGD [149], resulting in both reduced depressant effects on synaptic transmission and accelerated recovery (Fig. **3**). Electrophysiological data obtained recently *in vivo* involving delays in the time to ECoG suppression during repeated global cerebral ischemia and kainic acid-induced seizures are consistent with the concept of adenosine depletion following metabolic stress [84]. Furthermore, prolonged cerebral ischemia results in waning adenosine release *in vivo* in cat cortex [129], whilst *in vitro*, Ca^2+^-free medium resulted in increased adenosine release and exaggerated adenosine depletion [152].

We have postulated [148] that adenosine depletion may be injurious to the mammalian brain during repeated or subsequent insults to the brain through both reduced cerebral vasodilatation and reduced inhibition of excitatory synaptic transmission. This is dramatically emphasised by the return of the fEPSP to 50% of control 37 mins after the first OGD episode and only 15 mins after the second [149] (Fig. **3**), and may in part account for the increased vulnerability of the mammalian brain to repeated insults over 1 - 4 hours [99].

The basis of this loss of adenosine is not clear. It may involve prolonged depression of ATP levels in the post hypoxic/ischemic state (eg [67,100]), which may be exacerbated by the loss of downstream metabolites into the perfusate *in vitro*, and into the systemic circulation, as has been observed in humans following cerebral ischemia during carotid endarterectomy [211], stroke and transient ischemic attack [108]. These latter studies raise the interesting possibility that adenosine may serve as a biomarker for acute stroke or raised intracranial pressure.

**Fig. (4):**
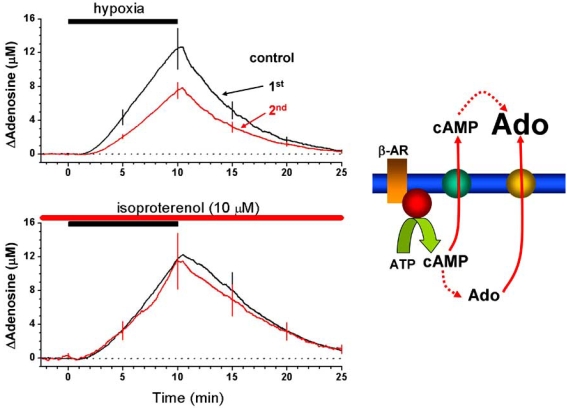
Restoration of adenosine release from a hippocampal slice by application of the β-adrenoceptor agonist isoproterenol during the second hypoxic period. Right panel, putative scheme by which stimulation of β-adrenoceptors may increase extracellular adenosine (ado). Modified from [151].

However, we have described a number of manipulations which either restore hypoxic/OGD release and/or the depressants effects of hypoxia. These include: a) allowing hippocampal slices to recover for two hours between episodes; b) application of exogenous adenosine; c) application of the β-adrenoceptor agonist isoproterenol (Fig. **4**) [151,152]. These observations imply that the "pool" of adenosine can be replenished with demonstrable functional effects. Potentially this might be exploited for therapeutic benefit in individuals vulnerable to repeated or secondary cerebral insults.

## SENSING BELOW THE SURFACE - MICROELECTRODE BIOSENSORS

**Fig. (5):**
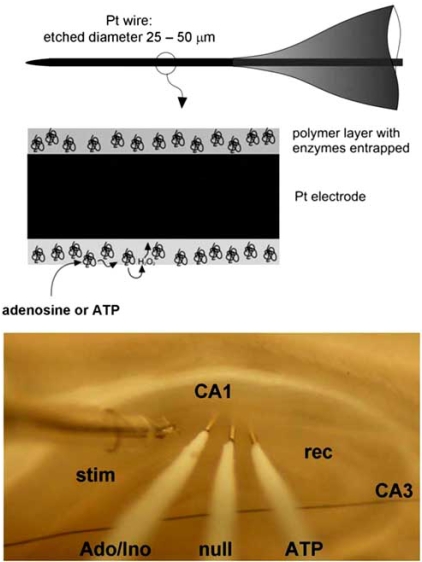
MK II sensor design (top) and use in hippocampal slices (bottom).

Subsequent refinements in sensor technology saw the immobilization of the adenosine metabolising enzymes on thin (25 - 50 μm) Pt/Ir wire [120] (Fig. **5**). This allowed insertion of the sensors into the hippocampal slice and obviated concerns [112] that surface recordings of adenosine release were somehow influenced by a layer of dead surface tissue. Conclusions drawn from the MKII sensors were practically the same as per surface measurements [65], but additionally revealed: a) An IC_50_ of around 1 μM for adenosine against the fEPSP during hypoxia; b) a surge of adenosine release on reoxygenation; c) hysteresis between the effects of adenosine on the fEPSP during the onset and recovery from hypoxia.

Estimates of adenosine concentration in the extracellular space range from the tens to low hundreds of nanomolar. The lower estimates obtained *in vivo* reflect either cortical cup sampling or recovery of tissue damage after insertion of microdialysis probes [154]. However, attempts to measure basal adenosine with MKII sensors inserted into hippocampal slices have not been conclusive as yet [66].

This may reflect the present requirement of subtracting an inosine signal from that of the adenosine sensor to yield a net, differential, signal. Subtle differences in extracellular adenosine may confound such spatially-disparate measurements. An alternative is the application of the ecto-ATPase inhibitor ARL 67156 which reduced the adenosine tone by around 110 nM [66]. This latter observation is similar to that obtained by Dulla *et al.,* using MKI adenosine sensors in the presence of ARL 67156 [45] and is consistent with the concept of an adenosine tone being provided by ATP release from astrocytes [146]. Estimates of extracellular adenosine have been made using pharmacological approaches, (180 – 240 nM, [111]; 140 – 200 nM; [47]), and HPLC techniques (264 nM; [168]) are in agreement with those estimated by the sensor using ARL 67156.

In the cerebellum the regulation of the extracellular purine tone is unexpectedly complex [208]. This tone can vary from one region to another. Often the tone can be measured (with biosensors) as inosine or hypoxanthine rather than as adenosine. Nevertheless an adenosine tone is usually apparent as a tonic inhibition of parallel fibre purkinje cell synapses. This raises the notion that there may be synaptic and non-synaptic extracellular compartments of adenosine.

Why these *in vitro* values of basal adenosine are elevated compared to some, but not all, obtained *in vivo* [154] may reflect the reduced recovery of adenosine during *in vivo* sampling or the potential for inadequate oxygenation of *in vitro* brain slices [105].

Estimates provided by the adenosine sensor for the efficacy of endogenous adenosine against synaptic transmission during hypoxia give an IC_50_ value of around 0.45 - 1.25 μM [65,149,151] and values of 20 - 30 μM for peak adenosine concentrations achieved during hypoxia/OGD [149]. These values are close to those inferred pharmacologically for the IC_50_ of exogenous adenosine against transmission of 600 - 760 nM [47], and peak OGD values *in vitro* [111], and measured directly, albeit acutely after probe insertion, *in vivo* [51,74].

**Fig. (6):**
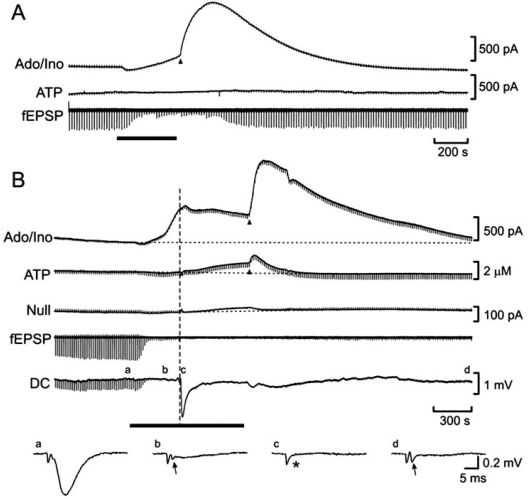
MK II sensor measurements of adenosine and ATP release during *in vitro* ischemia. **A**) Adenosine release increases gradually during the *in vitro* ischemic episode (black bar) then displays a surge on reoxygenation (black arrowhead). Short episodes of OGD elicit no ATP release. **B**) Longer episodes provoke the anoxic depolarisation and elicit ATP release, which also displays a surge on reoxygenation. Modified from [66].

Continuous sampling of extracellular adenosine during hypoxia or OGD with adenosine sensors inserted into the hippocampal slice have revealed two phases of adenosine release (Fig. **6**): the first is a gradual accumulation during the episode and a second, usually much larger surge (the post-hypoxic/OGD purine efflux; PPE) on reoxygenation [65,66] not readily apparent with surface measurements [34,65,149,151,152]. This maybe due to smoothing of the adenosine signal along the (horizontal) length of the sensor as opposed to sampling from a more restricted quasi-vertical tissue column when inserted into the slice.

It is interesting that this surge occurs when synaptic transmission is recovering in the slice - giving rise to the hysteresis we observe between the IC_50_ of adenosine against transmission during the onset and recovery [65,149]. Given this, it is unlikely that the function of this surge is to suppress glutamatergic excitation in the immediate post-hypoxic/OGD phase, since this occurs when transmission is either severely depressed by prolonged OGD or inhibited by omission of Ca^2+^ or addition of TTX or glutamate receptor antagonists [65]. Instead, this adenosine surge may give rise, *via* A_2A_Rs, to vasodilatation and the hyperemia observed in the cerebral circulation in response to hypoxia or ischemia [133].

As to why the fEPSP recovers in the face of this large surge of adenosine was discussed in detail by Pearson *et al.,* [149] where the IC_50_ for fEPSP suppression during the onset of hypoxia was 0.46 μM and increased over 10-fold to 5.7 μM during the recovery phase. Homologous or heterologous desensitisation/inhibition by A_1_, A_2A_ and A_3_Rs was ruled out, leaving the possibility that additional neurotransmitters/modulators may participate in the initial depression phase, thereby decreasing the apparent IC_50_ for adenosine against the fEPSP. However, focal deletion [4], knockout [89] and pharmacological antagonism [57,58,71] of A_1_Rs leave little scope for additional inhibitory agents during hypoxia/OGD. Potentially, microdomains of adenosine, caused by localised changes in adenosine release or metabolism, beyond the resolution of adenosine sensors may give rise to this phenomenon.

## ATP RELEASE

The release of adenosine during metabolic stress has now been investigated in a number of systems and is clearly released during the very early stages of hypoxia/ischemia. Questions then arise as to whether ATP is released under these conditions and whether ATP is the precursor to adenosine.

There is pharmacological, immunohistochemical and biochemical evidence that suggests that ATP is released in response to ischemia. For example, protein and activity levels of some ecto-nucleotidases, the enzymes responsible for the extracellular metabolism of ATP [231], were increased post-ischemia [13] and pharmacological antagonists of P2 receptors are generally protective under such conditions [62]. These observations are consistent with ATP release in response to cerebral metabolic stress.

Attempts have been made to measure ATP release during hypoxia/ischemia using microdialysis and cortical cup techniques. In one study hypoxia failed to elevate extracellular nucleotide levels, despite a doubling of adenosine release, suggesting that adenosine was released as such and not as a nucleotide precursor [159]. However, more severe insults *in vitro* [90] and *in vivo* [132] have elicited demonstrable ATP release from rat hippocampal slices and rat striatum, respectively. These latter studies do not inform as to the temporal sequence of release events and whether ATP is released before adenosine or vice versa. Attempts to resolve this have been made in the anoxia-resistant turtle brain [124]. In this microdialysis/HPLC/bioluminescence study, both adenosine and ATP release was delayed until 60 min after the onset of anoxia. The authors felt it unlikely that ATP release contributed to that of adenosine since the concentration of ATP was two orders of magnitude lower than those of adenosine, and ATP release was observed to increase during extended anoxia whilst the levels of adenosine declined (the latter being reminiscent of findings in the mammalian brain).

To address, with enhanced temporal resolution the release of ATP and adenosine during metabolic stress in the mammalian brain, we have recently used ATP sensors [121], simultaneously with adenosine sensors, during OGD in hippocampal slices [66]. As per previous findings we observed rapid elevations in extracellular adenosine coincident with the depression of the fEPSP. However, no ATP was observed during short OGD episodes (Fig. **6A**). Furthermore, the surge of adenosine on rexoygenation was not accompanied by a rise in extracellular ATP, suggesting that the adenosine surge was not dependent on ATP release.

In contrast, ATP release was invariably observed when the slice experienced the anoxic depolarization (AD; Fig. **6B**). Furthermore, once the AD had occurred, ATP also showed a surge on reoxygenation. Thus, from the time just prior to the AD, extracellular ATP rose from a low level of ~100 nM to reach ~700 nM at reoxygenation (some 1 - 2 mins after the AD) and peaked at 1800 nM during the post-OGD surge. As might be predicted, the ecto-ATPase inhibitor ARL 67156 potentiated all phases of ATP release, particularly the post-OGD surge, but had no bearing on adenosine release.

**Fig. (7):**
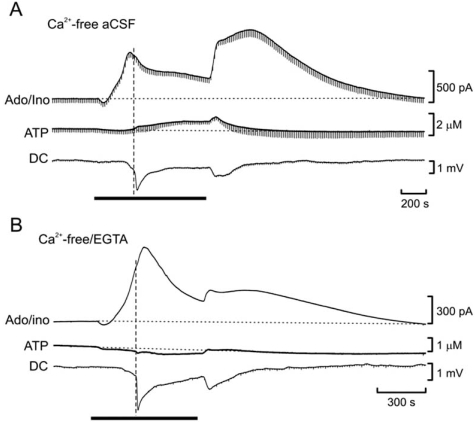
Differential dependency of adenosine and ATP release on extracellular Ca^2+^. **A**) Release of adenosine during in vitro ischemia (black bar) is enhanced in nominally Ca^2+^-free aCSF, but ATP release is unaffected. **B**) Addition of EGTA to nominally Ca^2+^-free aCSF prevents ATP but not adenosine release. Modified from [66].

ATP release was inseparable from the AD: the AD was delayed in younger animals, as was ATP release, whilst the AD was accelerated in Ca^2+^-free medium, as was ATP release (Fig. **7**). Whole-cell patch-clamp recordings confirmed the timing of ATP release *vs* the AD. An interesting question is whether ATP release precedes the AD or vice versa. That the AD occurs in Ca^2+^-free/EGTA- containing aCSF, but ATP release is prevented (Fig. **7**), suggests that the AD precipitates ATP release and that this release occurs by some process with very high affinity for extracellular Ca^2+^. Confirmation of the relative timing between ATP release and *in vivo* spreading depression has recently been provided by an independent group using ATP sensors [182]. In this study KCl-induced spreading depression evoked large (100 μM) ATP transients in rat neocortex.

Is this ATP release a harbinger of impending neuronal death? Not necessarily, at least not over the life-time of *in vitro* brain slice studies: ATP release occurred during conditions that would allow the recovery of synaptic transmission after *in vitro* ischemia – eg NMDA receptor antagonism, in the presence of TTX and in the absence of extracellular Ca^2+^ [66]. Thus, ATP release does not necessarily reflect cellular lysis, but instead reflects a process activated by, or associated with, strong (anoxic or spreading) depolarisation. Furthermore, Schock *et al.,* (2007) concluded that ATP release and activation of P2 receptors may contribute to protective ischemic tolerance [40,41]. In contrast, work from Maiken Nedergaard’s lab suggests that ATP (measured in culture using luciferin/luciferase) is released during hypoxic preconditioning which is then metabolised to adenosine (measured in culture using adenosine sensors) to initiate the preconditioning cascade *via* A_2B_Rs *in vitro* and A_1_Rs *in vivo* [114]. Regardless of whether ATP or adenosine are mediators of ischemic preconditioning, it is clear that the release of ATP may be an important factor in post-ischemic survival of brain tissue.

In summary, the rapid hypoxic/ischemic release of adenosine, and delayed release of ATP, subserve disparate functions in the mammalian brain. In terms of adenosine release, it is likely that this release is important for the inhibition of excitatory synaptic transmission and may contribute to the acute neuroprotective efficacy of adenosine [29]. Further dilatory actions of adenosine on the cerebrovasculature [106] serve to maximise the delivery of blood to compromised tissue. In contrast, the outcomes of ATP release, which may be associated with spreading-depression, may either have deleterious effects *per se*, in terms of activation of pore-forming receptors, or may offset these damaging consequences through initiation, either directly or indirectly, of ischemic preconditioning.

## ADENOSINE RELEASE DURING SEIZURE ACTIVITY

It has been known for many years that seizures cause large decreases in cerebral ATP [101]. Around the time when McIlwain showed that electrical stimulation caused accumulations of cAMP and release of adenosine in brain slices, Sattin demonstrated that seizure activity caused accumulation of cAMP in brain tissue *in vivo* [179]. As might be expected on the basis of his earlier work, caffeine and theophylline greatly reduced this accumulation, but precipitated more severe seizures - an early indication of the anticonvulsant actions of endogenous adenosine. Subsequent work revealed elevations in tissue adenosine *in vivo* in response to electrical stimulation [174], with the expectation that it would be released into the extracellular space.

Although rapid elevations of whole brain and cortical adenosine levels were observed during seizure activity induced by electrical stimulation [185] and bicuculline [184, 220,221], attempts by Schrader and colleagues [184] to measure increased adenosine efflux into the superior sagittal sinus were unsuccessful. A subsequent HPLC study by Berne and colleagues did reveal increases in interstitial adenosine following bicuculline-induced seizures in newborn piglets, but not when attempts were made to increase the oxygen supply to the brain *via* increased mean arterial blood pressure and increased partial pressure of oxygen (pO_2_) of the dialysis perfusate [145]. This suggests that hypoxia associated with seizure activity may be an important determinant of seizure-induced adenosine release. However, pharmacological and adenosine sensor experiments show that adenosine release occurs *in vitro* in response to very brief (10 - 15 s) seizures under conditions where oxygenation, of the medium at least, remains constant [54,55]. This does not preclude that there are very localised synaptic changes in oxygenation favouring ATP breakdown and adenosine accumulation and release [203]. What may distinguish between the two (O_2_-dependent and O_2_-independent release) are differential mechanisms in adenosine release under the two conditions.

Adenosine release into the interstitial fluid during seizures has also been demonstrated in humans [50]. In this seminal study, During and Spencer bilaterally implanted microdialysis probes into the hippocampi of 4 patients being prepped for ablative surgery for intractable complex partial epilepsy. Measurements of adenosine release were made over 10 - 16 days during which time at least 3 spontaneous seizures were recorded for each patient.

Basal adenosine levels, corrected for recovery and 3 - 5 days after probe insertion, measured 2.33 μM (89 nM uncorrected) in the non-epileptogenic hippocampus and 1.8 μM (67 nM uncorrected) in the epileptogenic hippocampus. It is tempting to speculate that the lower levels of adenosine in the epileptogenic hippocampus reflects astrogliosis-mediated increase in adenosine kinase expression and activity, which would lower extracellular adenosine levels and also lower seizure threshold [9]. During seizures extracellular adenosine rose dramatically in the ipsilateral hippocampus by between 7.5 and 31-fold, whilst in the contralateral hippocampus adenosine rose by 5.8 - 8.4 fold. The elevation in the epileptogenic hippocampus persisted for 18 mins after the seizure which prompted the authors to conclude that adenosine was an important factor, not only in seizure termination, but also in the refractory period after seizures during which the threshold for seizures is increased whilst the duration is decreased. Electrophysiological evidence for this contention has been provided and reviewed by significant figures in the field such as Dragunow [42,43] and Dunwiddie [46], and indeed from humans where caffeine, aminophylline/theophylline and hyperventilation (see below) are used to prolong seizure activity during electroconvulsive therapy [35].

As an interesting historical footnote, in a final test in these patients towards the end of the study, the microdialysis probe was flushed with 56 mM K^+^ for 30 mins, in the presence and absence of Ca^2+^ in the medium. In the presence of extracellular Ca^2+^ KCl evoked a large ~4-fold increase in adenosine, which was greatly attenuated by the removal of Ca^2+^. Ca^2+^-free medium itself caused a small increase in dialysate adenosine (from ~60 nM to ~90 nM), reminiscent of earlier work indicative of an inhibitory influence of Ca^2+^ on adenosine release. Subsequent microdialysis studies *in vivo* in rat hippocampus confirmed that seizures evoked by a variety of means (bicuculline, kainic acid or pentylenetetrazol) caused the release of adenosine, as well as the metabolites inosine, hypoxanthine and xanthine [7].

The functions of this adenosine release range from causing cerebral vasodilatation and ictal hyperemia *via* A_2_ receptors [230] and suppression of neuronal excitation *via* A_1_ receptors [46].

In a pharmacological study, we were able to show that the rapid inhibition of the fEPSP induced by seizures recorded from 600 μm hippocampal slices perfused with Mg^2+^-free aCSF was due to the activation of A_1_ receptors [54]. Whereas seizures evoked by brief electrical stimulation (2 s, 60 Hz) typically lasted for ~20 s, the fEPSP took several minutes to recover, indicative of a form of post-ictal refractoriness. Although application of an A_1_R antagonist resulted in a doubling of seizure duration and increase in seizure intensity, the inhibition of the fEPSP was greatly attenuated, suggesting an important role for endogenous adenosine in seizure suppression and indeed in limiting the spread of seizures as observed in A_1_R KO mice [56]. In this study we were able to show that the activation of A_2A_ and A_3_Rs had a proconvulsant effect through the use of selective antagonists which attenuated seizure duration [54]. This raises the possibility that the potential use of A_2A_R antagonists may extend beyond Parkinson's Disease and into seizure disorders [113].

**Fig. (8):**
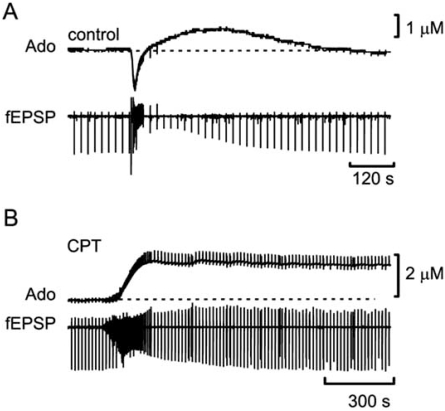
Release of adenosine during **A**) brief electrically-evoked seizures and **B**) spontaneous seizures in the presence of the A1R antagonist CPT. Periodic downward deflections reflect fEPSPs evoked at 15 s intervals. Following seizure activity the fEPSP is transiently depressed - less so in CPT, despite a much longer seizure. Modified from [55].

More recently, using adenosine biosensors we have been able to visualise directly the release of adenosine during evoked and spontaneous seizure activity (Fig. **8**) [55]. We found that electrically-evoked seizures lasting ~10 s were associated with the release of ~1 μM adenosine, which remained elevated for around 4 - 5 min, a time course similar to the recovery of the fEPSP. Application of the A_1_R antagonist 8-CPT elicited intense spontaneous seizures of around 50 - 60 s duration and peak adenosine release of 2 μM. Under these conditions extracellular adenosine was elevated for prolonged periods, most likely reflecting frequent spontaneous seizures and increased interictal activity.

## ATP RELEASE DURING SEIZURES

Of course, as per hypoxic/ischemic adenosine release, these observations of adenosine release during seizure activity beg the question as to whether or not ATP is released as a precursor to adenosine.

Early indications that adenine nucleotides were released by depolarisation and electrical stimulation of brain slices [78,163,186] were given a boost in the mid 1970s following the introduction of a luciferin-luciferase technique for the direct demonstration of ATP release [86]. In this preparation, an extract of firefly lanterns was dripped over slices of the electric organ of *Torpedo marmorata*, against which a photomultiplier tube was pressed. Electrical stimulation of the electric nerve resulted in increases in emitted light, which could be blocked by curare and potentiated by eserine. Using this technique Thomas White showed the direct release of ATP from a synaptosomal preparation in response to stimulation by KCl and veratridine, the former of which was sensitive to extracellular Ca^2+^, whilst the latter was blocked by TTX [214].

Evidence that ATP release occurred in response to electrical stimulation *in vivo* was obtained by John Phillis from somatosensory cortex using luciferin-luciferase. In these experiments, ATP release was not sampled on-line, but samples from the cortical cup were taken at intervals and showed large elevations in ATP in response to direct stimulation of the cortex [223]. These studies were extended by Wieraszko *et al.,* who used luciferin-luciferase to reveal the direct release of ATP during stimulation of the Schaffer pathway in hippocampal slices, which was Ca^2+^ dependent, but not dependent on, or induced by, glutamate receptor activation, suggesting presynaptic release of ATP [216].

Notwithstanding the concerns of Hamann and Attwell of ATP release caused by stimulation-induced electroporation of axons [76], these studies showing the release of ATP in a manner consistent with neurotransmitters presaged the demonstrations of synaptic currents mediated by ATP in medial habenula [52], hippocampus [135,142] and neocortex [143] and in other structures such as locus coeruleus [139] and dorsal horn of the spinal cord [6] (for a review see [3,95]). Indeed, a vesicular ATP transporter (VNUT1) has recently been described [181]. These observations suggest that there may be an ATP and P2 component in the neuronal hyperexcitability of seizure states, but this has not been conclusively addressed.

There have been suggestions that ATP is proconvulsant *via* P2X receptors, since the excitatory actions of α, β-meATP in a low Mg^2+^/4-AP hippocampal slice model of seizure activity was reduced by suramin and PPADS [173]. This is consistent with the presence and stimulatory actions of P2X receptors (P2X_1_, P2X_2/3_, and P2X_3_) on glutamate release in hippocampal synaptosomes, whereas P2Y_1_, P2Y_2_, and P2Y_4_ receptors inhibit glutamate release [171]. Under these conditions the enhanced release of ATP, as determined with luciferin-luciferase, in seizure prone DBA/2 mice [217], might contribute to seizure propensity, as might upregulation of P2X_7_ receptors [206], although this latter observation is not universal [94].

In contrast, down-regulation of P2X_2_ and P2X_4_ receptors have been described in seizure-sensitive gerbil hippocampus [93], as has a decrease in evoked ATP release in kindled hippocampal synaptosomes [168]. Changes in the evoked release of ATP in seizure states are reflected in alterations in ecto-nucleotidase activity [10,11,137,140,168,183], although these may be developmentally-regulated [36]. A number of recent reviews cover the potential role of ATP in seizure activity [61,107].

A recent publication suggested that the release of endogenous ATP and adenosine is influenced by extracellular pH (pHe) [45], which is known to change during seizure activity [187]. In this study, hypercapnia (10 - 20 % CO_2_) caused pHe to drop and a depression of the fEPSP. This was associated with an increase in extracellular adenosine, as measured with MKI sensors on the surface of the slice. The depression of transmission was mediated in part by both A_1_Rs and P2 receptors since only the combination of DPCPX (A_1_Rs) and PPADS (P2Rs) fully prevented the inhibition of the fEPSP. Conversely, hypocapnia (2 % CO_2_), which caused tissue alkalosis, resulted in an enhancement of the fEPSP. This enhancement was mostly occluded by the excitation produced by A_1_R antagonism. However, a small excitatory role for P2Rs was also discerned under these conditions. Similar observations on hypercapnia were made in spinal cord, although intracellular acidification was considered responsible for the release of adenosine [141].

That CO_2_ levels might have a bearing on seizure activity is evidenced by the fact that hyperventilation, which reduces CO_2_ and induces hypocapnia, is used to prolong electroconvulsive seizures [35] and to elicit absence, ie thalamocortical, [136] and temporal lobe seizures [72] in vulnerable individuals. Accordingly, Dulla *et al.,* [45] showed that hypocapnia (2 % CO_2_) caused a decrease in extracellular adenosine and an increase in interictal burst frequency in area CA3, which was occluded by DPCPX and the P2 antagonist suramin. Thus, certain forms of seizures may have a strong purinergic component manifest by the interaction of both adenosine and ATP. Subsequent studies with microelectrode ATP biosensors may reveal this putative ATP component.

## MECHANISMS OF PURINE RELEASE

The question as to how and from where the purines adenosine and ATP are released is complex. The answer will depend critically upon the nature of the stimulus and may potentially involve multiple temporally- and spatially-overlapping sources from several cellular sites. This is especially the case for adenosine and ATP given their ubiquitous distribution across all cell types.

Having said that, an important role of astrocytes in adenosine release during hypoxia/OGD has been suggested through the use of pure astrocyte cultures, which release adenosine during hypoxia/OGD, or the gliotoxin fluorocitrate in hippocampal slices, which prevented the hypoxic depression of the fEPSP to a similar extent to the A_1_R antagonist CPT, suggesting that intact glial metabolism is required for adenosine release during hypoxia [125]. In a similar vein, the acute depression of synaptic transmission by the fluorocitrate precursor fluoroacetate was reversed by A_1_R antagonism [26].

However, the mechanism by which hypoxic/OGD adenosine release occurs is not clear. For example, inhibition of equilibrative nucleoside transporters (ENTs; [98]) with currently-available drugs does not prevent the release of adenosine during hypoxia/OGD *in vitro* [66,125,152] (and may indeed potentiate release), a condition where one might reasonably expect the elevation of intracellular adenosine to be expelled *via* ENTs.

Alternatively, additional mechanisms of purine efflux exist that have been described since the initial descriptions of vesicular- and transporter-mediated release, which may play profound roles in regulating the availability of extracellular adenosine. Given that a considerable body of literature exists on "traditional" purine conduits [49,68,112,215], we now focus our attention on more recent additions to the panoply of purine release mechanisms.

## REVISITING THE MECHANISMS OF ADENOSINE RELEASE:

### Exocytosis

A common consensus is that adenosine arises in the extracellular space either through the breakdown of ATP, or through the action of equilibrative transporters. Nevertheless there is mounting evidence for other mechanisms of adenosine release some of which resemble exocytotic release (see [210] for a review).

In the cerebellum adenosine release can be evoked by brief tetanic stimulation of the parallel fibres by a mechanism that is both Ca^2+^-dependent and TTX-sensitive, two common hallmarks of exocytotic release [209]. This release of adenosine occurs in the apparent absence of ATP release, cannot be altered by compounds that inhibit ectonucleotidases, is insensitive to transport inhibitors and does not depend on activation of glutamate receptors. The likeliest explanation is that it represents release of adenosine from vesicles. Nevertheless, this a controversial conclusion and further definitive evidence is required to eliminate the possibility of any other type of mechanism before the notion of exocytotic release of adenosine will gain widespread acceptance.

### Retrograde Signalling

An interesting concept proposed by Tom Dunwiddie was that adenosine could be released from neurones in an antidromic fashion, from the postsynaptic dendrite to the presynaptic terminal, thereby regulating neurotransmitter release in response to postsynaptic activity. This suggestion of retrograde signalling presaged the explosion of interest in retrograde signalling by endocannabinoids and other transmitters such as GABA and glutamate. Brundege and Dunwiddie [15] showed that loading postsynaptic CA1 pyramidal neurones with exogenous adenosine resulted in a larger suppression of synaptic transmission, as defined by the greater effects of adenosine receptor antagonists and adenosine deaminase on synaptic transmission, and a raised paired-pulse facilitation ratio. However, attempts to elevate *endogenous* adenosine in single neurones sufficiently to influence synaptic function were not successful [17]. Nonetheless, these studies leaving open the tempting suggestion that neuronal or glial retrograde signalling by adenosine or ATP [85,178] might still be a fruitful avenue to pursue in hippocampus and other brain regions [77].

## MECHANISMS OF ATP RELEASE

While ATP is undoubtedly present in, and released from, vesicles through exocytosis [3,144], channel-mediated release also seems to be important under certain circumstances.

### Release Through Gap Junction Hemichannels

There are two gene families capable of forming hemichannels and gap junctions (the docking of hemichannels in two opposed membranes to form an intercellular pathway): the connexins and pannexins [188]. Pannexins are homologous to the invertebrate innexins. Gap junctions are well known to be permeable to molecules of a molecular weight of up to around 1000. Thus the idea that ATP and other small metabolites could permeate gap junctions has a long history. However, for a long time it was not apparent that hemichannels had any physiological role.

The first suggestion that hemichannels could be important in their own right came from studies on cultured astrocytes, which suggested a role for Cx43-mediated ATP release in the propagation of Ca^2+^ waves elicited by mechanical stimulation [191,192]. Nevertheless there was resistance to the idea that hemichannels could gate open under normal circumstances. Further reports followed demonstrating hemichannel-mediated ATP release in the developing cortex [212] and retina [147], increasing acceptance of the idea that hemichannels could release ATP under physiological circumstances. Hemichannel mediated-ATP release and associated P2Y receptor-generated Ca^2+^ signalling seems to be a common mechanism in several developmental contexts [32]. However, neither A_1_R nor P2Rs seem to participate in astrocytic Ca^2+^ waves that develop over a period of an hour following photothrombosis-induced ischemia [39].

Elegant studies from Nedergaard’s group have demonstrated directly ATP release through Cx43 hemichannels in isolated membrane patches [92]. As astrocytes possess Cx43 hemichannels this leaves open the possibility that astrocytes can release ATP by this route, but this is controversial. For example Bowser and Khakh [12] present evidence that propagating Ca^2+^ waves in astrocytes occur *via* exocytotic release of ATP, which is consistent with the evidence from Pascual *et al.,* [146] which suggests that ATP release from astrocytes depends upon SNARE-dependent membrane fusion. Interestingly, a small delay in the initial hypoxic depression of the fEPSP was observed in dominant-negative SNARE mice suggesting a contribution of this process to extracellular adenosine during the early stages of hypoxia [125]. Nevertheless Cx43-mediated ATP release from astrocytes could play a role in pathophysiological events such as responses to hypoxia [114] (however see [66] and [125] and below for countervailing views).

Meanwhile, Dahl and colleagues found that pannexin-1 could form ATP-permeable hemichannels. Initially this was demonstrated as a response to mechanical stimulation [5]. These authors also found that pannexin-1 gating could be modulated by intracellular Ca^2+^ [123] and that ATP could act back to inhibit pannexin-1 gating [164]. Evidence that ATP release through pannexin-1 hemichannels can be of physiological significance comes from the demonstration that they release ATP from taste receptor cells to activate afferent transmission [81,172].

Thompson *et al.,* [199] demonstrated that large conductance channels open in hippocampal neurons during OGD. As these channels were permeable to fluorescent dyes and could be blocked by hemichannel inhibitors they suggested that the channels were hemichannels, possibly pannexin-1. This pannexin channel gating could give rise to ATP release. However Frenguelli *et al.,* [66] demonstrated that ATP release during OGD was insensitive to carbenoxolone, an effective blocker of connexin and pannexin hemichannels, and Martin *et al.,* [125] were similarly unable to block adenosine release with the gap junction blockers flufenamic acid and 18α-glycyrrhetinic acid. Nonetheless, connexion gap junctions and hemichannels have been implicated in ischemic cell death [28,169].

Pannexin-1 hemichannels are present in pyramidal cell dendrites. Their gating, triggered by NMDA receptor activation, contributes to seizure generation in hippocampal neurons [198] raising the possibility that ATP release *via* this route could play a role in epileptiform activity.

### Other Ion Channels

A range of other channels have been postulated as conduits for ATP release (reviewed by Sabirov and Okada [177]). These include-volume activated anion channels, CFTR, the maxi anion channel [176] and the P2X_7_ receptor itself [193]. However, the P2X_7_ receptor does not seem to contribute to hypoxic adenosine release from cultured astrocytes [125] and the volume-activated anion channel blockers tamoxifen and DNDS did not affect the (largely adenosine-dependent) hypoxic depression of the fEPSP [150].

The difficulty in this field is that many of the pharmacological tools required to identify the involvement of particular channels lack useful selectivity to discriminate between them [190]. A range of experimental data is required, including direct measurement through patch clamp recording of ATP-permeant channel gating and the use of genetic methods, such as siRNA knockdown, to confirm definitively the molecular identity of the channel.

The evidence for ATP-permeation of the maxi-anion channel is particularly convincing [176]. Recent evidence implicates this channel in the release of ATP from cultured astrocytes during osmotically induced swelling [119] and OGD [118]. Whether this pathway of ATP release is significant *in vivo* remains to be determined.

In summary there are many competing claims with regard to the mechanisms of ATP release from cells. There are likely to be multiple pathways by which ATP is released and this complexity of release may be especially evident under pathophysiological conditions. Nevertheless, simplification of our understanding of ATP release may arise from the rigorous use of more selective pharmacological tools and genetic methods to dissect the relevant pathways.

## FUTURE PERSPECTIVES

### Future Developments in Purine Sensing

The MKI sensor [31] was an advance in terms of temporal and spatial resolution of extracellular adenosine measurements. Even then (late 1990s/early 2000s) it was clear that there was a need for greater spatial and temporal resolution, as well as the requirement for sensing within, as opposed to from the surface, of tissue. Many of these issues were addressed by the MKII microelectrode sensors [120, 121]. However, particularly with the explosion of interest in neuroglial signalling, there is a desire to make measurements from individual cells and potentially from individual release sites. That these high spatio/temporal requirements may be met by future developments in sensor design is a distinct possibility thereby providing devices that can be placed adjacent to cells/release sites to register the release of minute quantities of purines and other transmitters. Parallel developments in fast cyclic voltammetry of adenosine [27,82,197] may yield sub-second temporal resolution for adenosine as it has done for dopamine [218]. Genetics may provide a means of targeting purine-responsive proteins to specific cells and sites. These proteins, engineered to respond to extracellular adenosine, ATP and other neuroactive compounds, through changes in fluorescence intensity or wavelength, may provide the ultimate in spatial and temporal resolution.

### Future Developments in Purine Signalling

Purine research has matured enormously in the past 80 years - so much so that it now has an 'ome of its own - the Purinome [207]. However, many issues remain outstanding - including the cellular sources and mechanisms of purine release under conditions - for example seizures and ischemia - that likely recruit a panoply of cell types and purine conduits. Thus, we are further forward in defining cells capable of mediating release and novel release mechanisms, but the temporal sequence and relative contribution of each has yet to be determined.

This information is important if we are to harness the enormous potential of the Purinome as a therapeutic target: directing enhanced adenosine release to specific areas, *via* adenosine kinase inhibition for example, may promote protection *via* P1 receptors, whilst discerning protective *vs* damaging aspects of P2 receptors may yield specific targets for selective intervention in a range of acute and chronic neurological conditions.

Such an understanding will however only come about through greater insight into the complexities of purinergic signalling. This will come about *via* 1) greater availability of selective agonists and antagonists, particularly for P2 receptors, 2) selective blockers of the myriad conduits for the release of adenosine, ATP and other endogenous purinergic agonists and 3) electrochemical and genetic techniques with high spatio-temporal resolution for the release of purines from discrete cellular locations.

Realising many of these challenges is within sight: purine research has had a welcome shot in the arm through the role of purines in gliotransmission, connexins/pannexins as purine channels, and the realisation that the widespread (side) effects of purines described by Drury & Szent-Györgyi can be circumvented, creating a growing base of purine-based medicines [23,87]. The ensuing influx of new investigators bring with them new techniques, new questions and new demands, which will hopefully be met by parallel advances in pharmacology, optogenetics and amperometry.

The future has never looked brighter for purinology. Let's hope that the next eighty years see as remarkable advance in our understanding as the previous eighty have.

## References

[1] Abbracchio MP, Boeynaems JM, Barnard EA, Boyer JL, Kennedy C, Miras-Portugal MT, King BF, Gachet C, Jacobson KA, Weisman GA, Burnstock G (2003). Characterization of the UDP-glucose receptor (re-named here the P2Y14 receptor) adds diversity to the P2Y receptor family. Trends Pharmacol. Sci.

[2] Abbracchio MP, Burnstock G, Boeynaems JM, Barnard EA, Boyer JL, Kennedy C, Knight GE, Fumagalli M, Gachet C, Jacobson KA, Weisman GA (2006). International Union of Pharmacology LVIII: update on the P2Y G protein-coupled nucleotide receptors: from molecular mechanisms and pathophysiology to therapy. Pharmacol. Rev.

[3] Abbracchio MP, Burnstock G, Verkhratsky A, Zimmermann H (2009). Purinergic signalling in the nervous system: an overview. Trends Neurosci.

[4] Arrigoni E, Crocker AJ, Saper CB, Greene RW, Scammell TE (2005). Deletion of presynaptic adenosine A_1_ receptors impairs the recovery of synaptic transmission after hypoxia. Neuroscience.

[5] Bao L, Locovei S, Dahl G (2004). Pannexin membrane channels are mechanosensitive conduits for ATP. FEBS Lett.

[6] Bardoni R, Goldstein PA, Lee CJ, Gu JG, MacDermott AB (1997). ATP P2X receptors mediate fast synaptic transmission in the dorsal horn of the rat spinal cord. J. Neurosci.

[7] Berman RF, Fredholm BB, Aden U, O'Connor WT (2000). Evidence for increased dorsal hippocampal adenosine release and metabolism during pharmacologically induced seizures in rats. Brain Res.

[8] Berne RM, Rubio R, Curran GH (1974). Release of adenosine from ischemic brain. Effect on cerbral vasculature resistance and incorporation into cerebral adenine nucleotides. Circ. Res.

[9] Boison D (2008). The adenosine kinase hypothesis of epileptogenesis. Prog. Neurobiol.

[10] Bonan CD, Amaral OB, Rockenbach IC, Walz R, Battastini AM, Izquierdo I, Sarkis JJ (2000). Altered ATP hydrolysis induced by pentylenetetrazol kindling in rat brain synaptosomes. Neurochem. Res.

[11] Bonan CD, Walz R, Pereira GS, Worm PV, Battastini AM, Cavalheiro EA, Izquierdo I, Sarkis JJ (2000). Changes in synaptosomal ectonucleotidase activities in two rat models of temporal lobe epilepsy. Epilepsy Res.

[12] Bowser DN, Khakh BS (2007). Vesicular ATP is the predominant cause of intercellular calcium waves in astrocytes. J. Gen. Physiol.

[13] Braun N, Zhu Y, Krieglstein J, Culmsee C, Zimmermann H (1998). Upregulation of the enzyme chain hydrolyzing extracellular ATP after transient forebrain ischemia in the rat. J. Neurosci.

[14] Brown P, Dale N (2000). Adenosine A_1_ receptors modulate high voltage-activated Ca^2+^ currents and motor pattern generation in the xenopus embryo. J. Physiol.

[15] Brundege JM, Dunwiddie TV (1996). Modulation of excitatory synaptic transmission by adenosine released from single hippocampal pyramidal neurons. J. Neurosci.

[16] Brundege JM, Dunwiddie TV (1997). Role of adenosine as a modulator of synaptic activity in the central nervous system. Adv. Pharmacol.

[17] Brundege JM, Dunwiddie TV (1998). Metabolic regulation of endogenous adenosine release from single neurons. Neuroreport.

[18] Brust TB, Cayabyab FS, MacVicar BA (2007). C-Jun N-terminal kinase regulates adenosine A_1_ receptor-mediated synaptic depression in the rat hippocampus. Neuropharmacology.

[19] Brust TB, Cayabyab FS, Zhou N, MacVicar BA (2006). p38 mitogen-activated protein kinase contributes to adenosine A_1_ receptor-mediated synaptic depression in area CA1 of the rat hippocampus. J. Neurosci.

[20] Burnstock G (1972). Purinergic nerves. Pharmacol. Rev.

[21] Burnstock G (2006). Historical review: ATP as a neurotransmitter. Trends Pharmacol. Sci.

[22] Burnstock G (2007). Physiology and pathophysiology of purinergic neurotransmission. Physiol. Rev.

[23] Burnstock G (2008). Purinergic signalling and disorders of the central nervous system. Nat. Rev. Drug Discov.

[24] Burnstock G, Campbell G, Satchell D, Smythe A (1970). Evidence that adenosine triphosphate or a related nucleotide is the transmitter substance released by non-adrenergic inhibitory nerves in the gut. Br. J. Pharmacol.

[25] Burnstock G, Kennedy C (1985). Is there a basis for distinguishing two types of P2-purinoceptor?. Gen. Pharmacol.

[26] Canals S, Larrosa B, Pintor J, Mena MA, Herreras O (2008). Metabolic challenge to glia activates an adenosine-mediated safety mechanism that promotes neuronal survival by delaying the onset of spreading depression waves. J. Cereb. Blood Flow Metab.

[27] Cechova S, Venton BJ (2008). Transient adenosine efflux in the rat caudate-putamen. J. Neurochem.

[28] Contreras JE, Sanchez HA, Veliz LP, Bukauskas FF, Ben-nett MV, Saez JC (2004). Role of connexin-based gap junction channels and hemichannels in ischemia-induced cell death in nervous tissue. Brain Res. Brain Res. Rev.

[29] Cunha RA (2005). Neuroprotection by adenosine in the brain: From A1 receptor activation to A2A blockade. Purinerg. Signal.

[30] Curtis DR, Phillis JW, Watkins JC (1961). Cholinergic and non-cholinergic transmission in the mammalian spinal cord. J. Physiol.

[31] Dale N (1998). Delayed production of adenosine underlies temporal modulation of swimming in frog embryo. J. Physiol.

[32] Dale N (2008). Dynamic ATP signalling and neural development. J. Physiol.

[33] Dale N, Gourine AV, Llaudet E, Bulmer D, Thomas T, Spyer KM (2002). Rapid adenosine release in the nucleus tractus solitarii during defence response in rats: real-time measurement *in vivo*. J. Physiol.

[34] Dale N, Pearson T, Frenguelli BG (2000). Direct measurement of adenosine release during hypoxia in the CA1 region of the rat hippocampal slice. J. Physiol.

[35] Datto C, Rai AK, Ilivicky HJ, Caroff SN (2002). Augmentation of seizure induction in electroconvulsive therapy: a clinical reappraisal. J. ECT.

[36] de Paula CG, Bruno AN, Vuaden FC, Sarkis JJ, Bonan CD (2005). Ontogenetic profile of ectonucleotidase activities from brain synaptosomes of pilocarpine-treated rats. Int. J. Dev. Neurosci.

[37] Deuticke B, Gerlach E, Dierkesmann R (1966). [Decomposition of free nucleotides in the rat heart, skeletal muscle, brain and liver in oxygen deficiency]. Pflugers Arch. Gesamte Physiol. Menschen Tiere.

[38] DiGeronimo RJ, Gegg CA, Zuckerman SL (1998). Adenosine depletion alters postictal hypoxic cerebral vasodilation in the new-born pig. Am. J. Physiol.

[39] Ding S, Wang T, Cui W, Haydon PG (2009). Photothrombosis ischemia stimulates a sustained astrocytic Ca2+ signaling *in vivo*. Glia.

[40] Dirnagl U, Meisel A (2008). Endogenous neuroprotection: mitochondria as gateways to cerebral preconditioning?. Neuropharmacology.

[41] Dirnagl U, Simon RP, Hallenbeck JM (2003). Ischemic tolerance and endogenous neuroprotection. Trends Neurosci.

[42] Dragunow M (1986). Adenosine: the brains natural anticonvulsant?. Trends Pharmac. Sci.

[43] Dragunow M, Goddard GV, Laverty R (1987). Proconvulsant effects of theophylline on hippocampal afterdischarges. Exp. Neurol.

[44] Drury AN, Szent-Gyorgyi A (1929). The physiological activity of adenine compounds with especial reference to their action upon the mammalian heart. J. Physiol.

[45] Dulla C, Dobelis P, Pearson T, Frenguelli BG, Staley KJ, Masino SA (2005). Adenosine and ATP link P_CO2_ to cortical excitability via pH. Neuron.

[46] Dunwiddie TV (1999). Adenosine and suppression of seizures. Adv. Neurol.

[47] Dunwiddie TV, Diao L (1994). Extracellular adenosine concentrations in hippocampal brain slices and the tonic inhibitory modulation of evoked excitatory responses. J. Pharmacol. Exp. Ther.

[48] Dunwiddie TV, Hoffer BJ (1980). Adenine nucleotides and synaptic transmission in the *in vitro* rat hippocampus. Br. J. Pharmacol.

[49] Dunwiddie TV, Masino SA (2001). The role and regulation of adenosine in the central nervous system. Annu. Rev. Neurosci.

[50] During MJ, Spencer DD (1992). Adenosine: a potential mediator of seizure arrest and postictal refractoriness. Ann. Neurol.

[51] Dux E, Fastbom J, Ungerstedt U, Rudolphi K, Fredholm BB (1990). Protective effect of adenosine and a novel xanthine derivative propentofylline on the cell damage after bilateral carotid occlusion in the gerbil hippocampus. Brain Res.

[52] Edwards FA, Gibb AJ, Colquhoun D (1992). ATP receptor-mediated synaptic currents in the central nervous system. Nature.

[53] Egan TM, Samways DS, Li Z (2006). Biophysics of P2X receptors. Pflugers Arch.

[54] Etherington LA, Frenguelli BG (2004). Endogenous adenosine modulates epileptiform activity in rat hippocampus in a receptor subtype-dependent manner. Eur. J. Neurosci.

[55] Etherington LA, Patterson GE, Meechan L, Boison D, Irving AJ, Dale N, Frenguelli BG (2009). Astrocytic adenosine kinase regulates basal synaptic adenosine levels and seizure activity but not activity-dependent adenosine release in the hippocampus. Neuropharmacology.

[56] Fedele DE, Li T, Lan JQ, Fredholm BB, Boison D (2006). Adenosine A_1_ receptors are crucial in keeping an epileptic focus localized. Exp. Neurol.

[57] Fowler JC (1989). Adenosine antagonists delay hypoxia-induced depressions of neuronal activity in hippocampal brain slice. Brain Res.

[58] Fowler JC (1990). Adenosine antagonists alter the synaptic response to *in vitro* ischemia in the rat hippocampus. Brain Res.

[59] Fowler JC (1993). Changes in extracellular adenosine levels and population spike amplitude during graded hypoxia in the rat hippocampal slice. Naunyn Schmiedebergs Arch. Pharmacol.

[60] Fowler JC (1993). Purine release and inhibition of synaptic transmission during hypoxia and hypoglycemia in rat hippocampal slices. Neurosci. Lett.

[61] Franke H, Illes P (2006). Involvement of P2 receptors in the growth and survival of neurons in the CNS. Pharmacol. Ther.

[62] Franke H, Krugel U, Illes P (2006). P2 receptors and neuronal injury. Pflugers Arch.

[63] Fredholm BB, Dunwiddie TV, Bergman B, Lindstrom K (1984). Levels of adenosine and adenine nucleotides in slices of rat hippocampus. Brain Res.

[64] Fredholm BB, IJzerman AP, Jacobson KA, Klotz KN, Lin-den J (2001). International Union of Pharmacology. XXV. Nomenclature and classification of adenosine receptors. Pharmacol. Rev.

[65] Frenguelli BG, Llaudet E, Dale N (2003). High-resolution real-time recording with microelectrode biosensors reveals novel aspects of adenosine release during hypoxia in rat hippocampal slices. J. Neurochem.

[66] Frenguelli BG, Wigmore G, Llaudet E, Dale N (2007). Temporal and mechanistic dissociation of ATP and adenosine release during ischemia in the mammalian hippocampus. J. Neurochem.

[67] Gadalla AE, Pearson T, Currie AJ, Dale N, Hawley SA, Sheehan M, Hirst W, Michel AD, Randall A, Hardie DG, Frenguelli BG (2004). AICA riboside both activates AMP-activated protein kinase and competes with adenosine for the nucleoside transporter in the CA1 region of the rat hippocampus. J. Neurochem.

[68] Geiger JD, Fyda DM, Stone T.W (1991). Adenosine transport in nervous system tissues. Adenosine in the nervous system.

[69] Gervitz LM, Lutherer LO, Davies DG, Pirch JH, Fowler JC (2001). Adenosine induces initial hypoxic-ischemic depression of synaptic transmission in the rat hippocampus *in vivo*. Am. J. Physiol. Regul. Integr. Comp. Physiol.

[70] Gordon GR, Choi HB, Rungta RL, Ellis-Davies GC, MacVicar BA (2008). Brain metabolism dictates the polarity of astrocyte control over arterioles. Nature.

[71] Gribkoff VK, Bauman LA, VanderMaelen CP (1990). The adenosine antagonist 8-cyclopentyltheophylline reduces the depression of hippocampal neuronal responses during hypoxia. Brain Res.

[72] Guaranha MS, Garzon E, Buchpiguel CA, Tazima S, Yacubian EM, Sakamoto AC (2005). Hyperventilation revisited: physiological effects and efficacy on focal seizure activation in the era of video-EEG monitoring. Epilepsia.

[73] Gundlfinger A, Bischofberger J, Johenning FW, Torvinen M, Schmitz D, Breustedt J (2007). Adenosine modulates transmission at the hippocampal mossy fibre synapse via direct inhibition of presynaptic calcium channels. J. Physiol.

[74] Hagberg H, Andersson P, Lacarewicz J, Jacobson I, Butcher S, Sandberg M (1987). Extracellular adenosine, inosine, hypoxanthine, and xanthine in relation to tissue nucleotides and purines in rat striatum during transient ischemia. J. Neurochem.

[75] Halassa MM, Florian C, Fellin T, Munoz JR, Lee SY, Abel T, Haydon PG, Frank MG (2009). Astrocytic modulation of sleep homeostasis and cognitive consequences of sleep loss. Neuron.

[76] Hamann M, Attwell D (1996). Non-synaptic release of ATP by electrical stimulation in slices of rat hippocampus, cerebellum and habenula. Eur. J. Neurosci.

[77] Harvey J, Lacey MG (1997). A postsynaptic interaction between dopamine D1 and NMDA receptors promotes presynaptic inhibition in the rat nucleus accumbens via adenosine release. J. Neurosci.

[78] Heller IH, McIlwain H (1973). Release of (14 C)adenine derivatives from isolated subsystems of the guinea pig brain: actions of electrical stimulation and of papaverine. Brain Res.

[79] Holton FA, Holton P (1953). The possibility that ATP is a transmitter at sensory nerve endings. J. Physiol.

[80] Huang M, Daly JW (1974). Adenosine-elicited accumulation of cyclic AMP in brain slices: potentiation by agents which inhibit uptake of adenosine. Life Sci.

[81] Huang YJ, Maruyama Y, Dvoryanchikov G, Pereira E, Chaudhari N, Roper SD (2007). The role of pannexin 1 hemichannels in ATP release and cell-cell communication in mouse taste buds. Proc. Natl. Acad. Sci. USA.

[82] Huffman ML, Venton BJ (2009). Carbon-fiber microelectrodes for in vivo applications. Analyst.

[83] Ilie A, Ciocan D, Zagrean AM, Nita DA, Zagrean L, Moldovan M (2006). Endogenous activation of adenosine A^1^ receptors accelerates ischemic suppression of spontaneous electrocortical activity. J. Neurophysiol.

[84] Ilie A, Spulber S, Avramescu S, Nita DA, Zagrean AM, Zagrean L, Moldovan M (2006). Delayed ischemic electrocortical suppression during rapid repeated cerebral ischemia and kainate-induced seizures in rat. Eur. J. Neurosci.

[85] Israel M, Lesbats B, Manaranche R, Meunier FM, Frachon P (1980). Retrograde inhibition of transmitter release by ATP. J. Neurochem.

[86] Israel M, Lesbats B, Meunier FM, Stinnakre J (1976). Postsynaptic release of adenosine triphosphate induced by single impulse transmitter action. Proc. R. Soc. Lond. B. Biol. Sci.

[87] Jacobson KA, Gao ZG (2006). Adenosine receptors as therapeutic targets. Nat. Rev. Drug Discov.

[88] Jarvis MF, Khakh BS (2009). ATP-gated P2X cation-channels. Neuropharmacology.

[89] Johansson B, Halldner L, Dunwiddie TV, Masino SA, Poel-chen W, Gimenez-Llort L, Escorihuela RM, Fernandez-Teruel A, Wiesenfeld-Hallin Z, Xu XJ, Hardemark A, Betsholtz C, Herlenius E, Fredholm BB (2001). Hyperalgesia, anxiety, and decreased hypoxic neuroprotection in mice lacking the adenosine A^1^ receptor. Proc. Natl. Acad. Sci. USA.

[90] Juranyi Z, Sperlágh B, Vizi ES (1999). Involvement of P2 purinoceptors and the nitric oxide pathway in [3H]purine outflow evoked by short-term hypoxia and hypoglycemia in rat hippocampal slices. Brain Res.

[91] Kakiuchi S, Rall TW, McIlwain H (1969). The effect of electrical stimulation upon the accumulation of adenosine 3',5'-phosphate in isolated cerebral tissue. J. Neurochem.

[92] Kang J, Kang N, Lovatt D, Torres A, Zhao Z, Lin J, Neder-gaard M (2008). Connexin 43 hemichannels are permeable to ATP. J. Neurosci.

[93] Kang TC, An SJ, Park SK, Hwang IK, Won MH (2003). P2X2 and P2X4 receptor expression is regulated by a GABA_A_ receptor-mediated mechanism in the gerbil hippocampus. Brain Res. Mol. Brain Res.

[94] Kang TC, Park SK, Hwang IK, An SJ, Won MH (2004). GABA_B_ receptor-mediated regulation of P2X_7_ receptor expression in the gerbil hippocampus. Brain Res. Mol. Brain Res.

[95] Khakh BS (2001). Molecular physiology of P2X receptors and ATP signalling at synapses. Nat. Rev. Neurosci.

[96] Khakh BS, North RA (2006). P2X receptors as cell-surface ATP sensors in health and disease. Nature.

[97] Kim M, Yu ZX, Fredholm BB, Rivkees SA (2005). Susceptibility of the developing brain to acute hypoglycemia involving A_1_ adenosine receptor activation. Am. J. Physiol. Endocrinol. Metab.

[98] King AE, Ackley MA, Cass CE, Young JD, Baldwin SA (2006). Nucleoside transporters: from scavengers to novel therapeutic targets. Trends Pharmacol. Sci.

[99] Kirino T (2002). Ischemic tolerance. J. Cereb. Blood Flow Metab.

[100] Kleihues P, Kobayashi K, Hossmann KA (1974). Purine nucleotide metabolism in the cat brain after one hour of complete ischemia. J. Neurochem.

[101] Klein JR, Olsen NS (1947). Effect of convulsive activity upon the concentration of brain glucose, glycogen, lactate, and phosphates. J. Biol. Chem.

[102] Kostopoulos GK, Limacher JJ, Phillis JW (1975). Action of various adenine derivatives on cerebellar Purkinje cells. Brain Res.

[103] Krnjevic K (2008). Electrophysiology of cerebral ischemia. Neuropharmacology.

[104] Krnjevic K, Phillis JW (1963). Actions of certain amines on cerebral cortical neurones. Br. J. Pharmacol. Chemother.

[105] Kukley M, Schwan M, Fredholm BB, Dietrich D (2005). The role of extracellular adenosine in regulating mossy fiber synaptic plasticity. J. Neurosci.

[106] Kulik T, Kusano Y, Aronhime S, Sandler AL, Winn HR (2008). Regulation of cerebral vasculature in normal and ischemic brain. Neuropharmacology.

[107] Kumaria A, Tolias CM, Burnstock G (2008). ATP signalling in epilepsy. Purinerg. Signal.

[108] Laghi-Pasini F, Guideri F, Picano E, Parenti G, Petersen C, Varga A, Di Perri T (2000). Increase in plasma adenosine during brain ischemia in man: a study during transient ischemic attacks, and stroke. Brain Res. Bull.

[109] Landolt HP (2008). Sleep homeostasis: a role for adenosine in humans?. Biochem. Pharmacol.

[110] Latini S, Bordoni F, Corradetti R, Pepeu G, Pedata F (1998). Temporal correlation between adenosine outflow and synaptic potential inhibition in rat hippocampal slices during ischemia-like conditions. Brain Res.

[111] Latini S, Bordoni F, Pedata F, Corradetti R (1999). Extracellular adenosine concentrations during *in vitro* ischaemia in rat hippocampal slices. Br. J. Pharmacol.

[112] Latini S, Pedata F (2001). Adenosine in the central nervous system: release mechanisms and extracellular concentrations. J. Neurochem.

[113] LeWitt PA, Guttman M, Tetrud JW, Tuite PJ, Mori A, Chaikin P, Sussman NM (2008). Adenosine A_2A_ receptor antagonist istradefylline (KW-6002) reduces "off" time in Parkinson's disease: a double-blind, randomized, multicenter clinical trial (6002-US-005). Ann. Neurol.

[114] Lin JH, Lou N, Kang N, Takano T, Hu F, Han X, Xu Q, Lovatt D, Torres A, Willecke K, Yang J, Kang J, Nedergaard M (2008). A central role of connexin 43 in hypoxic preconditioning. J. Neurosci.

[115] Lipton P, Robacker K (1982). Adenosine may cause an early inhibition of synaptic transmision during anoxia. Soc. Neurosci. Abstr.

[116] Lipton P, Whittingham TS (1982). Reduced ATP concentration as a basis for synaptic transmission failure during hypoxia in the *in vitro* hippocampal slice. J. Physiol.

[117] Lipton PS, Whittingham TS, Dingledine R (1984). Energy metabolism and brain slice function. Brain slices.

[118] Liu HT, Sabirov RZ, Okada Y (2008). Oxygen-glucose deprivation induces ATP release via maxi-anion channels in astrocytes. Purinerg. Signal.

[119] Liu HT, Toychiev AH, Takahashi N, Sabirov RZ, Okada Y (2008). Maxi-anion channel as a candidate pathway for osmosensitive ATP release from mouse astrocytes in primary culture. Cell Res.

[120] Llaudet E, Botting NP, Crayston JA, Dale N (2003). A three-enzyme microelectrode sensor for detecting purine release from central nervous system. Biosens. Bioelectron.

[121] Llaudet E, Hatz S, Droniou M, Dale N (2005). Microelectrode biosensor for real-time measurement of ATP in biological tissue. Anal. Chem.

[122] Lloyd HG, Spence I, Johnston GA (1988). Involvement of adenosine in synaptic depression induced by a brief period of hypoxia in isolated spinal cord of neonatal rat. Brain Res.

[123] Locovei S, Wang J, Dahl G (2006). Activation of pannexin 1 channels by ATP through P2Y receptors and by cytoplasmic calcium. FEBS Lett.

[124] Lutz PL, Kabler S (1997). Release of adenosine and ATP in the brain of the freshwater turtle (*Trachemys scripta*) during long-term anoxia. Brain Res.

[125] Martin ED, Fernandez M, Perea G, Pascual O, Haydon PG, Araque A, Cena V (2007). Adenosine released by astrocytes contributes to hypoxia-induced modulation of synaptic transmission. Glia.

[126] Martin J, Sutherland CJ, Zbrozyna AW (1976). Habituation and conditioning of the defense reactions and their cardiovascular components in cats and dogs. Pflugers Arch.

[127] Martin RL, Lloyd HGE, Cowan AI (1994). The early events of oxygen and glucose deprivation: Setting the scene for neuronal death?. Trends Neurosci.

[128] Matsumoto K, Graf R, Rosner G, Shimada N, Heiss WD (1992). Flow thresholds for extracellular purine catabolite elevation in cat focal ischemia. Brain Res.

[129] Matsumoto K, Graf R, Rosner G, Taguchi J, Heiss WD (1993). Elevation of neuroactive substances in the cortex of cats during prolonged focal ischemia. J. Cereb. Blood Flow Metab.

[130] McIlwain H, Rabin BR, Freedman RB (1971). Cyclic AMP and tissues of the brain. Effects of Drugs on Cellular Control Mechanisms.

[131] McIlwain H, Pull I (1972). Release of adenine derivatives on electrical stimulation of superfused tissues from the brain. J. Physiol.

[132] Melani A, Turchi D, Vannucchi MG, Cipriani S, Gianfriddo M, Pedata F (2005). ATP extracellular concentrations are increased in the rat striatum during *in vivo* ischemia. Neurochem. Int.

[133] Miekisiak G, Kulik T, Kusano Y, Kung D, Chen JF, Winn HR (2008). Cerebral blood flow response in adenosine 2a receptor knockout mice during transient hypoxic hypoxia. J. Cereb. Blood Flow Metab.

[134] Mogul DJ, Adams ME, Fox AP (1993). Differential activation of adenosine receptors decreases N-type but potentiates P-type Ca^2+^ current in hippocampal CA3 neurons. Neuron.

[135] Mori M, Heuss C, Gahwiler BH, Gerber U (2001). Fast synaptic transmission mediated by P2X receptors in CA3 pyramidal cells of rat hippocampal slice cultures. J. Physiol.

[136] Murphy JV, Dehkharghani F (1994). Diagnosis of childhood seizure disorders. Epilepsia.

[137] Nagy AK, Houser CR, Delgado-Escueta AV (1990). Synaptosomal ATPase activities in temporal cortex and hippocampal formation of humans with focal epilepsy. Brain Res.

[138] Newby AC (1984). Adenosine and the concept of retaliatory metabolites. Trends Biochem. Sci.

[139] Nieber K, Poelchen W, Illes P (1997). Role of ATP in fast excitatory synaptic potentials in locus coeruleus neurones of the rat. Br. J. Pharmacol.

[140] Oses JP, Viola GG, de Paula CG, Junior VH, Hansel G, Bohmer AE, Leke R, Bruno AN, Bonan CD, Bogo MR, Portela LV, Souza DO, Sarkis JJ (2007). Pentylenetetrazol kindling alters adenine and guanine nucleotide catabolism in rat hippocampal slices and cerebrospinal fluid. Epilepsy Res.

[141] Otsuguro K, Yamaji Y, Ban M, Ohta T, Ito S (2006). Involvement of adenosine in depression of synaptic transmission during hypercapnia in isolated spinal cord of neonatal rats. J. Physiol.

[142] Pankratov Y, Castro E, Miras-Portugal MT, Krishtal O (1998). A purinergic component of the excitatory postsynaptic current mediated by P2X receptors in the CA1 neurons of the rat hippocampus. Eur. J. Neurosci.

[143] Pankratov Y, Lalo U, Krishtal O, Verkhratsky A (2002). Ionotropic P2X purinoreceptors mediate synaptic transmission in rat pyramidal neurones of layer II/III of somato-sensory cortex. J. Physiol.

[144] Pankratov Y, Lalo U, Verkhratsky A, North RA (2006). Vesicular release of ATP at central synapses. Pflugers Arch.

[145] Park TS, van Wylen DG, Rubio R, Berne RM (1987). Interstitial fluid adenosine and sagittal sinus blood flow during bicuculline-seizures in newborn piglets. J. Cereb. Blood Flow Metab.

[146] Pascual O, Casper KB, Kubera C, Zhang J, Revilla-Sanchez R, Sul JY, Takano H, Moss SJ, McCarthy K, Haydon PG (2005). Astrocytic purinergic signaling coordinates synaptic networks. Science.

[147] Pearson RA, Dale N, Llaudet E, Mobbs P (2005). ATP released via gap junction hemichannels from the pigment epithelium regulates neural retinal progenitor proliferation. Neuron.

[148] Pearson T, Currie AJ, Etherington LA, Gadalla AE, Damian K, Llaudet E, Dale N, Frenguelli BG (2003). Plasticity of purine release during cerebral ischemia: clinical implications?. J. Cell. Mol. Med.

[149] Pearson T, Damian K, Lynas R, Frenguelli BG (2006). Sustained elevation of extracellular adenosine and activation of A_1_ receptors underlie the post-ischaemic inhibition of neuronal function in rat hippocampus *in vitro*. J. Neurochem.

[150] Pearson T, Frenguelli BG (2000). Volume-regulated anion channels do not contribute extracellular adenosine during the hypoxic  depression of excitatory synaptic transmission in area CA1 of rat hippocampus. Eur. J. Neurosci.

[151] Pearson T, Frenguelli BG (2004). Adrenoceptor subtype-specific acceleration of the hypoxic depression of excitatory synaptic transmission in area CA1 of rat hippocampus. Eur. J. Neurosci.

[152] Pearson T, Nuritova F, Caldwell D, Dale N, Frenguelli BG (2001). A depletable pool of adenosine in area CA1 of the rat hippocampus. J. Neurosci.

[153] Pedata F, Latini S, Pugliese AM, Pepeu G (1993). Investigations into the adenosine outflow from hippocampal slices evoked by ischemia-like conditions. J. Neurochem.

[154] Phillis JW (2004). Adenosine and adenine nucleotides as regulators of cerebral blood flow: roles of acidosis, cell swelling, and KATP channels. Crit. Rev. Neurobiol.

[155] Phillis JW, Kostopoulos GK (1975). Adenosine as a putative transmitter in the cerebral cortex. Studies with potentiators and antagonists. Life Sci.

[156] Phillis JW, Kostopoulos GK, Limacher JJ (1974). Depression of corticospinal cells by various purines and pyrimidines. Can. J. Physiol. Pharmacol.

[157] Phillis JW, Kostopoulos GK, Limacher JJ (1975). A potent depressant action of adenine derivatives on cerebral cortical neurones. Eur. J. Pharmacol.

[158] Phillis JW, O'Regan MH (2003). *In vivo* studies of the release of adenine 5 '-nucleotides, adenosine, and its metabolites from the rat brain. Drug Dev. Res.

[159] Phillis JW, O'Regan MH, Perkins LM (1993). Adenosine 5'-triphosphate release from the normoxic and hypoxic *in vivo* rat cerebral cortex. Neurosci. Lett.

[160] Proctor WR, Dunwiddie TV (1983). Adenosine inhibits calcium spikes in hippocampal pyramidal neurons *in vitro*. Neurosci. Lett.

[161] Pull I, McIlwain H (1972). Adenine derivatives as neurohumoral agents in the brain. The quantities liberated on excitation of superfused cerebral tissues. Biochem. J.

[162] Pull I, McIlwain H (1972). Metabolism of (14 C)adenine and derivatives by cerebral tissues, superfused and electrically stimulated. Biochem. J.

[163] Pull I, McIlwain H (1973). Output of (14C)adenine nucleotides and their derivatives from cerebral tissues. Tetrodotoxin-resistant and calcium ion-requiring components. Biochem. J.

[164] Qiu F, Dahl G (2009). A permeant regulating its permeation pore: inhibition of pannexin 1 channels by ATP. Am. J. Physiol. Cell. Physiol.

[165] Ralevic V, Burnstock G (1998). Receptors for purines and pyrimidines. Pharmacol. Rev.

[166] Rall TW, Sutherland EW (1958). Formation of a cyclic adenine ribonucleotide by tissue particles. J. Biol. Chem.

[167] Rathbone M, Pilutti L, Caciagli F, Jiang S (2008). Neurotrophic effects of extracellular guanosine. Nucleosides Nucleotides Nucleic Acids.

[168] Rebola N, Coelho JE, Costenla AR, Lopes LV, Parada A, Oliveira CR, Soares-Da-Silva P, de Mendonça A, Cunha RA (2003). Decrease of adenosine A_1_ receptor density and of adenosine neuromodulation in the hippocampus of kindled rats. Eur. J. Neurosci.

[169] Retamal MA, Schalper KA, Shoji KF, Orellana JA, Ben-nett MV, Saez JC (2007). Possible involvement of different connexin43 domains in plasma membrane permeabilization induced by ischemia-reperfusion. J. Membr. Biol.

[170] Roberts JA, Vial C, Digby HR, Agboh KC, Wen H, Atter-bury-Thomas A, Evans RJ (2006). Molecular properties of P2X receptors. Pflugers Arch.

[171] Rodrigues RJ, Almeida T, Richardson PJ, Oliveira CR, Cunha RA (2005). Dual presynaptic control by ATP of glutamate release via facilitatory P2X_1_, P2X_2/3_, and P2X_3_ and inhibitory P2Y_1_, P2Y_2_, and/or P2Y_4_ receptors in the rat hippocampus. J. Neurosci.

[172] Romanov RA, Rogachevskaja OA, Bystrova MF, Jiang P, Margolskee RF, Kolesnikov SS (2007). Afferent neurotransmission mediated by hemichannels in mammalian taste cells. EMBO J.

[173] Ross FM, Brodie MJ, Stone TW (1998). Modulation by adenine nucleotides of epileptiform activity in the CA3 region of rat hippocampal slices. Br. J. Pharmacol.

[174] Rubio R, Berne RM, Bockman EL, Curnish RR (1975). Relationship between adenosine concentration and oxygen supply in rat brain. Am. J. Physiol.

[175] Rudolphi KA, Schubert P, Parkinson FE, Fredholm BB (1992). Neuroprotective role of adenosine in cerebral ischaemia. Trends Pharmacol. Sci.

[176] Sabirov RZ, Dutta AK, Okada Y (2001). Volume-dependent ATP-conductive large-conductance anion channel as a pathway for swelling-induced ATP release. J. Gen. Physiol.

[177] Sabirov RZ, Okada Y (2005). ATP release via anion channels. Purinerg. Signal.

[178] Santos DA, Salgado AI, Cunha RA (2003). ATP is released from nerve terminals and from activated muscle fibres on stimulation of the rat phrenic nerve. Neurosci. Lett.

[179] Sattin A (1971). Increase in the content of adenosine 3',5'-monophosphate in mouse forebrain during seizures and prevention of the increase by methylxanthines. J. Neurochem.

[180] Sattin A, Rall TW (1970). The effect of adenosine and adenine nucleotides on the cyclic adenosine 3', 5'-phosphate content of guinea pig cerebral cortex slices. Mol. Pharmacol.

[181] Sawada K, Echigo N, Juge N, Miyaji T, Otsuka M, Omote H, Yamamoto A, Moriyama Y (2008). Identification of a vesicular nucleotide transporter. Proc. Natl. Acad. Sci. USA.

[182] Schock SC, Munyao N, Yakubchyk Y, Sabourin LA, Hakim AM, Ventureyra EC, Thompson CS (2007). Cortical spreading depression releases ATP into the extracellular space and purinergic receptor activation contributes to the induction of ischemic tolerance. Brain Res.

[183] Schoen SW, Ebert U, Loscher W (1999). 5'-Nucleotidase activity of mossy fibers in the dentate gyrus of normal and epileptic rats. Neuroscience.

[184] Schrader J, Wahl M, Kuschinsky W, Kreutzberg GW (1980). Increase of adenosine content in cerebral cortex of the cat during bicuculline-induced seizure. Pflugers Arch.

[185] Schultz V, Lowenstein JM (1978). The purine nucleotide cycle. Studies of ammonia production and interconversions of adenine and hypoxanthine nucleotides and nucleosides by rat brain *in situ*. J. Biol. Chem.

[186] Shimizu H, Creveling CR, Daly J (1970). Stimulated formation of adenosine 3',5'-cyclic phosphate in cerebral cortex: synergism between electrical activity and biogenic amines. Proc. Natl. Acad. Sci. USA.

[187] Siesjo BK, von Hanwehr R, Nergelius G, Nevander G, Ingvar M (1985). Extra- and intracellular pH in the brain during seizures and in the recovery period following the arrest of seizure activity. J. Cereb. Blood Flow Metab.

[188] Sohl G, Maxeiner S, Willecke K (2005). Expression and functions of neuronal gap junctions. Nat. Rev. Neurosci.

[189] Somjen GG (1980). Stimulus-evoked and seizure-related responses of extracellular calcium activity in spinal cord compared to those in cerebral cortex. J. Neurophysiol.

[190] Spray DC, Ye ZC, Ransom BR (2006). Functional connexin "hemichannels": a critical appraisal. Glia.

[191] Stout C, Goodenough DA, Paul DL (2004). Connexins: functions without junctions. Curr. Opin. Cell Biol.

[192] Stout CE, Costantin JL, Naus CC, Charles AC (2002). Intercellular calcium signaling in astrocytes via ATP release through connexin hemichannels. J. Biol. Chem.

[193] Suadicani SO, Brosnan CF, Scemes E (2006). P2X7 receptors mediate ATP release and amplification of astrocytic intercellular Ca^2+^ signaling. J. Neurosci.

[194] Sulakhe PV, Phillis JW (1975). The release of 3H-adenosine and its derivatives from cat sensorimotor cortex. Life Sci.

[195] Sutherland EW, Rall TW (1958). Fractionation and characterization of a cyclic adenine ribonucleotide formed by tissue particles. J. Biol. Chem.

[196] Suzuki T, Namba K, Tsuga H, Nakata H (2006). Regulation of pharmacology by hetero-oligomerization between A_1_ adenosine receptor and P2Y_2_ receptor. Biochem. Biophys Res. Commun.

[197] Swamy BE, Venton BJ (2007). Subsecond detection of physiological adenosine concentrations using fast-scan cyclic voltammetry. Anal. Chem.

[198] Thompson RJ, Jackson MF, Olah ME, Rungta RL, Hines DJ, Beazely MA, MacDonald JF, MacVicar BA (2008). Activation of pannexin-1 hemichannels augments aberrant bursting in the hippocampus. Science.

[199] Thompson RJ, Zhou N, MacVicar BA (2006). Ischemia opens neuronal gap junction hemichannels. Science.

[200] Tonazzini I, Trincavelli ML, Montali M, Martini C (2008). Regulation of A_1_ adenosine receptor functioning induced by P2Y_1_ purinergic receptor activation in human astroglial cells. J. Neurosci. Res.

[201] Tonazzini I, Trincavelli ML, Storm-Mathisen J, Martini C, Bergersen LH (2007). Co-localization and functional cross-talk between A_1_ and P2Y_1_ purine receptors in rat hippocampus. Eur. J. Neurosci.

[202] Turner CP, Seli M, Ment L, Stewart W, Yan H, Johansson B, Fredholm BB, Blackburn M, Rivkees SA (2003). A_1_ adenosine receptors mediate hypoxia-induced ventriculomegaly. Proc. Natl. Acad. Sci. USA.

[203] Turner DA, Foster KA, Galeffi F, Somjen GG (2007). Differences in O_2_ availability resolve the apparent discrepancies in metabolic intrinsic optical signals *in vivo* and *in vitro*. Trends Neurosci.

[204] Valtysson J, Persson L, Hillered L (1998). Extracellular ischaemia markers in repeated global ischaemia and secondary hypoxaemia monitored by microdialysis in rat brain. Acta Neurochir. (Wien).

[205] Veech RL, Harris RL, Veloso D, Veech EH (1973). Freeze-blowing: a new technique for the study of brain *in vivo*. J. Neurochem.

[206] Vianna EP, Ferreira AT, Naffah-Mazzacoratti MG, Sanabria ER, Funke M, Cavalheiro EA, Fernandes MJ (2002). Evidence that ATP participates in the pathophysiology of pilocarpine-induced temporal lobe epilepsy: fluorimetric, immunohistochemical, and Western blot studies. Epilepsia.

[207] Volonte C, D'Ambrosi N (2009). Membrane compartments and purinergic signalling: the purinome, a complex interplay among ligands, degrading enzymes, receptors and transporters. FEBS J.

[208] Wall MJ, Atterbury A, Dale N (2007). Control of basal extracellular adenosine concentration in rat cerebellum. J. Physiol.

[209] Wall MJ, Dale N (2007). Auto-inhibition of rat parallel fibre-Purkinje cell synapses by activity-dependent adenosine release. J. Physiol.

[210] Wall MJ, Dale N (2008). Activity-Dependent Release of Adenosine: A Critical Re-Evaluation of Mechanism. Curr. Neuropharmacol.

[211] Weigand MA, Michel A, Eckstein HH, Martin E, Bardenheuer HJ (1999). Adenosine: a sensitive indicator of cerebral ischemia during carotid endarterectomy. Anesthesiology.

[212] Weissman TA, Riquelme PA, Ivic L, Flint AC, Kriegstein AR (2004). Calcium waves propagate through radial glial cells and modulate proliferation in the developing neocortex. Neuron.

[213] Wendler CC, Amatya S, McClaskey C, Ghatpande S, Fred-holm BB, Rivkees SA (2007). A_1_ adenosine receptors play an essential role in protecting the embryo against hypoxia. Proc. Natl. Acad. Sci. USA.

[214] White TD (1977). Direct detection of depolarisation-induced release of ATP from a synaptosomal preparation. Nature.

[215] White TD, Hoehn K, Stone TW (1991). Release of adenosine and ATP from nervous tissue. Adenosine in the nervous system.

[216] Wieraszko A, Goldsmith G, Seyfried TN (1989). Stimulation-dependent release of adenosine triphosphate from hippocampal slices. Brain Res.

[217] Wieraszko A, Seyfried TN (1989). Increased amount of extracellular ATP in stimulated hippocampal slices of seizure prone mice. Neurosci. Lett.

[218] Wightman RM (2006). Detection technologies. Probing cellular chemistry in biological systems with microelectrodes. Science.

[219] Winn HR, Rubio R, Berne RM (1979). Brain adenosine production in the rat during 60 seconds of ischemia. Circ. Res.

[220] Winn HR, Welsh JE, Berne RM, Rubio R (1979). Changes in brain adenosine during bicuculline-induced seizures: effect of altered arterial oxygen tensions. Trans. Am. Neurol. Assoc.

[221] Winn HR, Welsh JE, Rubio R, Berne RM (1980). Changes in brain adenosine during bicuculline-induced seizures in rats. Effects of hypoxia and altered systemic blood pressure. Circ. Res.

[222] Wu LG, Saggau P (1994). Adenosine inhibits evoked synaptic transmission primarily by reducing presynaptic calcium influx in area CA1 of hippocampus. Neuron.

[223] Wu PH, Phillis JW (1978). Distribution and release of adenosine triphosphate in rat brain. Neurochem. Res.

[224] Yawo H, Chuhma N (1993). Preferential inhibition of omega-conotoxin-sensitive presynaptic Ca^2+^ channels by adenosine autoreceptors. Nature.

[225] Yoshioka K, Hosoda R, Kuroda Y, Nakata H (2002). Hetero-oligomerization of adenosine A1 receptors with P2Y1 receptors in rat brains. FEBS Lett.

[226] Yoshioka K, Saitoh O, Nakata H (2001). Heteromeric association creates a P2Y-like adenosine receptor. Proc. Natl. Acad. Sci. USA.

[227] Yoshioka K, Saitoh O, Nakata H (2002). Agonist-promoted heteromeric oligomerization between adenosine A_1_ and P2Y_1_ receptors in living cells. FEBS Lett.

[228] Zhu PJ, Krnjevic K (1993). Adenosine release is a major cause of failure of synaptic transmission during hypoglycaemia in rat hippocampal slices. Neurosci. Lett.

[229] Zhu PJ, Krnjevic K (1997). Adenosine release mediates cyanide-induced suppression of CA1 neuronal activity. J. Neurosci.

[230] Zimmermann A, Domoki F, Bari F (2008). Seizure-induced alterations in cerebrovascular function in the neonate. Dev. Neurosci.

[231] Zimmermann H (2006). Ectonucleotidases in the nervous system. Novartis Found. Symp.

